# Supplementary material for: In-vivo studies on Transitmycin, a potent Mycobacterium tuberculosis inhibitor
Source: PLoS One. 2023 Mar 3;18(3):e0282454. doi: 10.1371/journal.pone.0282454 (PMC9983862; doi:10.1371/journal.pone.0282454)

**Supporting information**

**S1 Table. Scheme of TR administration in BALB/c mice in MTD determination**

| **S.**  **No** | **Groups** | **Route of Administration** | **No. of Animals (Equal Male &**  **Female)** | **Dosemg/kg** | **Duration** | **Studyperiod** |
| --- | --- | --- | --- | --- | --- | --- |
| 1 | Group- I | IV | 12 | 0.15 | Single Exposure | 14  Days post TR  administr  ation |
| 2 | Group- II |  | 12 | 0.3 |  |  |
| 3 | Group-III | IV | 12 | 0.45 |  |  |

## S2 Table. Scheme of TR administration in Dunkin Hartley Guinea pigs in MTD study

| **S.No** | **Test**  **compound** | **No. of**  **Animals** | **Dose** | |
| --- | --- | --- | --- | --- |
|  |  |  | **mg/kg** | **mg/animal** |
| **1** | TC-I | 1M | 0.04 | 0.015 |
| **2** | TC-II | 2M | 0.025 | 0.01 |
| Duration of exposure: 3doses (0days, 2^nd^ day, 5^th^ day) by Intra-peritoneal (IP) | | | | |

## S3 Table. Scheme of TR administration in Sprague Dawley rats and WNIN Rats in MTD study

| **Route– IV** | | | | | |
| --- | --- | --- | --- | --- | --- |
| **S.No** | **Group** | | **No.of**  **Animals** | **Dose** | |
|  |  |  |  | **mg/kg** | **mg/animal** |
| **1** | VC | | 2(1M+1F) | 10% DMSO | |
| **2** | GroupI | | 2(1M+1F) | 0.025 | 0.005 |
| **3** | GroupII | | 2(1M+1F) | 0.05 | 0.01 |
| **4** | Group III | | 2(1M+1F) | 0.1 | 0.02 |
| **5** | GroupIV | | 2(1M+1F) | 0.2 | 0.04 |
| **6** | GroupV | | 2(1M+1F) | 0.25 | 0.05 |
| **7** | GroupVI | | 2(1M+1F) | 0.4 | 0.08 |
| **8** | GroupVII | | 2(1M+1F) | 0.5 | 0.1 |
| **9** | GroupVIII | | 2(1M+1F) | 0.6 | 0.12 |
| **10** | GroupIX | | 2(1M+1F) | 0.75 | 0.15 |
| **Route of administration: IM** | | | | | |
| 1 | | VC | 4(2M+2F) | 10% DMSO | |
| 2 | | GroupI | 4(2M+2F) | 0.025 | 0.005 |
| 3 | | GroupII | 4(2M+2F) | 0.05 | 0.01 |
| 4 | | GroupIII | 4(2M+2F) | 0.1 | 0.02 |
| Intravenous(IV) as a single dose, Intramuscular dose twice a week | | | | | |

## S4 Table. Basic Experimental Scheme for *in-vivo* TR anti-TB efficacy study in Guinea pigs.

| **Group** | **Treatment** |
| --- | --- |
| Early control | 50-100 CFU of *M. tuberculosis strain* by aerosol inhalation on Day zero (Infection), sacrifice and plate lung and spleen on Day 15 to estimate CFU (Group 1). |
| Late control | Infection, no treatment, estimation of CFU at day7^th^, 12^th^ and day15^th^ post-treatment initiation (Group 2). |
| Positive control | Combination of Isoniazid (INH) and Rifampicin (Rif) at recommended, human equivalent doses i.e. RIF (50 mg/ kg body wt.) and INH (30 mg/kg body wt), treatment start 15 days post-infection by oral gavage daily, estimation of CFU at day 7^th^, 12^t h^and at day 15^th^ post- treatment initiation after a wash -out period of 2 days (Group 3). |
| Negative control | Infection, treatment starts 15 days post-infection- oral gavage of erythromycin daily (Group 4), estimation of CFU at day 7^th^, 12^th,^ andday15^th^post-treatment initiation after awash-out period of 2days^.^ |
| Group 5, 6 and 7 | Infection, treatment starts 15 days post-infection—I/P administration of TR every alternate day @ 0.01 mg/Kg body wt (Group 5), 0.02 mg /kgbody wt (Group 6), and 0.04 mg/kg body wt (Group 7), estimation of CFU at day 7^th^, 12^th^ and day 15^th^ post-treatment initiation after a wash-out period of 2 days. |

##

**S5 Table. MICs of TR (µg ml^-1^) determined in wildtype clinical isolates of MTB**

| **Culture. No.** | **TR MIC (in µg ml^-1^)** |
| --- | --- |
| 1 | 5 |
| 2 | 5 |
| 3 | 5 |
| 4 | 5 |
| 5 | 5 |
| 6 | 5 |
| 7 | 5 |
| 8 | 5 |
| 9 | 5 |
| 10 | 5 |
| 11 | 5 |
| 12 | 5 |
| 13 | 5 |
| 14 | 5 |
| 15 | 5 |
| 16 | 5 |
| 17 | 5 |
| 18 | 10 |
| 19 | 5 |
| 20 | 5 |
| 21 | 5 |
| 22 | 5 |
| 23 | 5 |
| 24 | 5 |
| 25 | 5 |
| 26 | 5 |
| 27 | 5 |
| 28 | 5 |
| 29 | 5 |
| 30 | 5 |
| 31 | 5 |
| 32 | 5 |
| 33 | 5 |
| 34 | 5 |
| 35 | 5 |
| 36 | 5 |
| 37 | 5 |
| 38 | 5 |
| 39 | 5 |
| 40 | 5 |
| 41 | 10 |
| 42 | 5 |
| 43 | 5 |
| 44 | 5 |
| 45 | 5 |
| 46 | 10 |
| 47 | 5 |
| 48 | 5 |
| 49 | 5 |

MICs for 49 *Mycobacterium tuberculosis* isolates that were susceptible to all the anti-TB drugs were used for determining the MIC of Transitmycin (TR).

## S6 Table. *In-vitro* Anti-TB efficacy testing on TR analogs by LRP assay

| **S.NO** | **CODE** | **Percentage of inhibition (%)**  **(at 40 μg/ml)** |
| --- | --- | --- |
| 1 | TR | 98.4 |
| 2 | TR-NC6 | 0 |
| 3 | TR-NSUND | 25.07 |
| 4 | TR-R | 62.5 |
| 5 | TR-RB | 44.1 |
| 6 | F-NMe | 77.3 |
| 7 | C-tertbutyl | 0 |

## S7 Table. Pharmacokinetic values of TR in uninfected guinea pigs

The plasma samples from TR control group received from JALMA were processed and the PK values were given below.

| S. no | Lab No | ug/ml |
| --- | --- | --- |
| 1 | Sample 1 PK - 21.08.19 | - |
| 2 | Sample 2 PK - 21.08.19 | 0.05 |
| 3 | Sample 3 GP - 3 - 21.08.19 (4hrs) | 0.21 |
| 4 | Sample 4 GP - 4 - 21.08.19 (6hrs) | 0.10 |
| 5 | Sample 5 GP - 5 - 21.08.19 (8hrs) 6.30pm | 0.06 |
| 6 | Sample 6 GP - 6 - 21.08.19 (12hrs) 10.30pm | 0.10 |
| 7 | Sample 7 GP - 7 - 22.08.19 (24hrs) 10.30am | 0.09 |

## S8 Table. Molecular structure of TR and its close analogue of known molecules

| **Sl.No** | **Compound Name** | **Mol.wt** | **Structure** |
| --- | --- | --- | --- |
| 0. | Transitmycin | 1269.4g/mol | 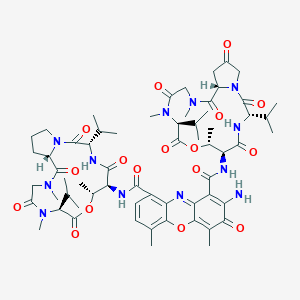 |
| 1. | Actinomycin D | 1255.4g/mol | 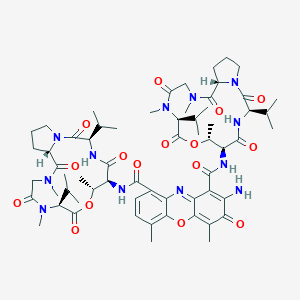 |
| 2. | 7-Amino-AMD | 1269.4g/mol | 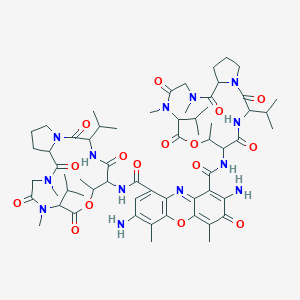 |
| 3. | Actinomycin C2 | 1269.4g/mol | 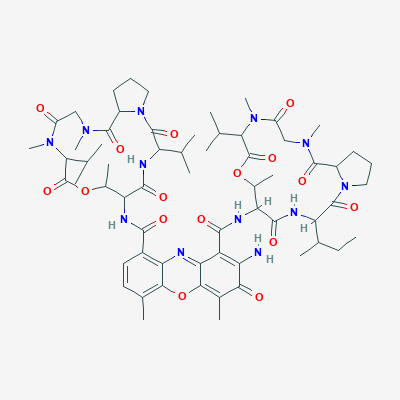 |
| 4. | Azetomycin II | 1227.4g/mol | 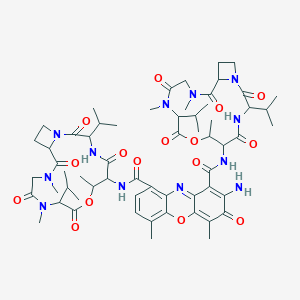 |
| 5. | Chloroactinom  ycin D | 1274.8g/mol | 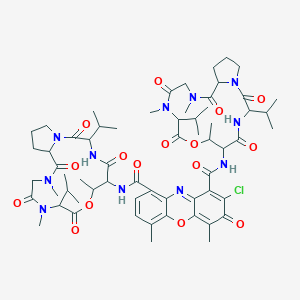 |
| 6. | Actinomycin C3 | 1283.5g/mol | 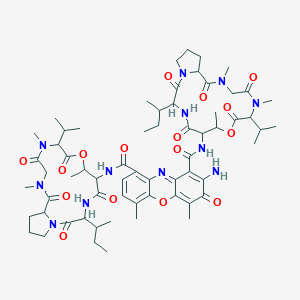 |
| 7. | Actinomycin X2 | 1269.4g/mol | 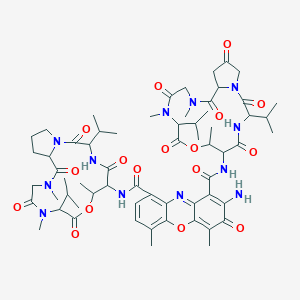 |
| 8. | Actinomycin D, 2A-D-leucine- | 1269.4g/mol | 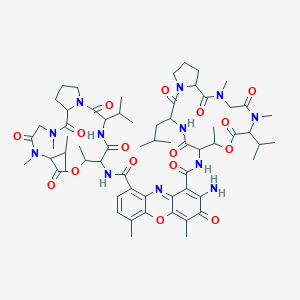 |
| 9. | Actinomycin A3 | 1265.4g/mol | 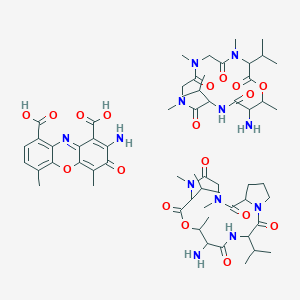 |
| 10. | Actinomycin F4 | 1257.4g/mol | 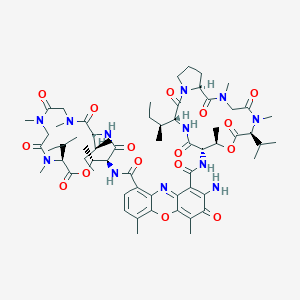 |
| 11. | Actinomycinxoa | 1271.4g/mol | 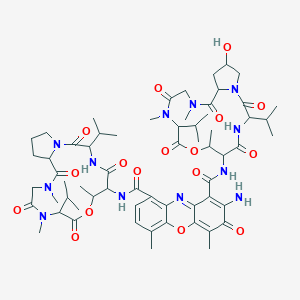 |
| 12. | CActinomycin | 1269.4g/mol | 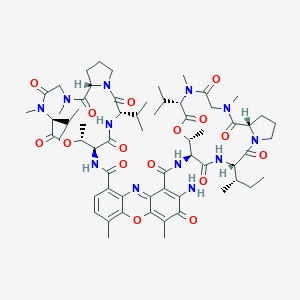 |
| 13. | Actinomycin VII | 1283.5g/mol | 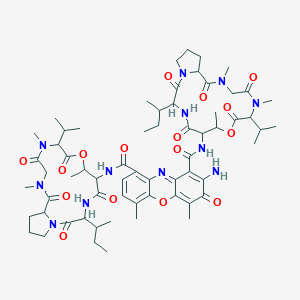 |
| 14. | Actinomycin P 2 | 1283.5g/mol | 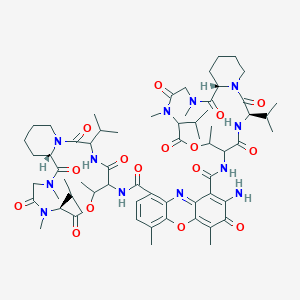 |
| 15. | 7-hydroxyactinomycin D | 1271.4g/mol | 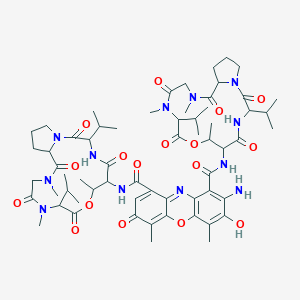 |

## S9 Table. Results of docking–TR chromophore with and without peptide, in the minor groove of DNA(G-C Region)

| Sl  .  no | Molecule name | Target Nam e | Docking score with(Chromophores) | Glide g score with(Chromophores) | glide model(chromophores) | Docking score with (Peptid  es) | Glide g score with(Peptide  s) | Glide model(Peptides) |
| --- | --- | --- | --- | --- | --- | --- | --- | --- |
| 1 | TR | 1MN  V | -9.839 | -9.839 | -110.393 | -6.045 | -6.045 | -76.063 |

## S10 Table. Results of docking-ActinomycinD chromophore with and without peptide.

| Sl.no | Moleculename | TargetName | Docking score with (Chromophores) | Glide g score with(Chromophores) | Glide model (chromophores) | Docking score with (Peptides) | Glide g score with (Peptid  es) | Glide model(Peptide) |
| --- | --- | --- | --- | --- | --- | --- | --- | --- |
| 1 | Acinomyc  inD | 1M  NV | -9.839 | -9.839 | -110.3 | -8.74 | -8.74 | -90.536 |

**S11 Table. Mortality during treatment with 0.01, 0.02, 0.04 kg^-1^ of TR in guinea pigs**

| Date | TR (0.01mg kg^-1^) | TR (0.02mg kg^-1^) | TR (0.04mg kg^-1^) |
| --- | --- | --- | --- |
| 6^th^-day post-treatment initiation  (After the third dose of TR) | No death | 1 death | 3 death |
| 7^th^-day post-treatment initiation  (After the third dose of TR) | No death | 2 death | 2 death |
| 8^th^-day post-treatment initiation  (After the third dose of TR) | No death | 2 death | 1 death |
| 11^th^-day post-treatment initiation  (After the fifth dose of TR) | One death |  |  |
| 12^th^-day post-treatment initiation  (After the sixth dose of TR) | One death |  |  |
| 13^th^-day post-treatment initiation  (After the sixth dose of TR) | One death |  |  |
| **The total no. of guinea pig died** | **3/6** | **5/6** | **6/6** |

##

**S1 Fig.** **Dosing schedule for safety study of TR in guinea pigs @0.001mg/kg.**

**S2 Fig. Timeline for drug efficacy evaluation using the guinea pig model of TB.**

**S3 Fig. Body weight of guinea pigs (numbered G1, G2 and G3 respectively) following treatment with TR @ 0.001mg kg-1 for one month. The guinea pig (G3) was sacrificed 3 days later after the last dose while G2 was sacrificed 15 days and G1 was monitored till 45 days after the last dose of TR before sacrifice.**

**
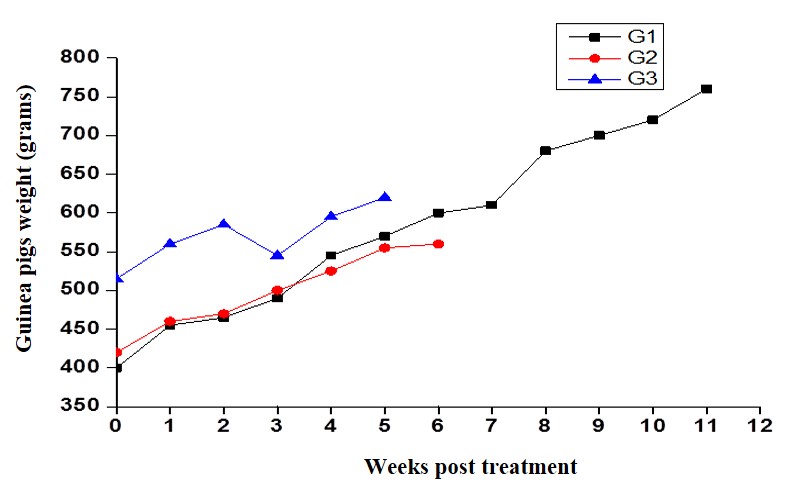
**

**
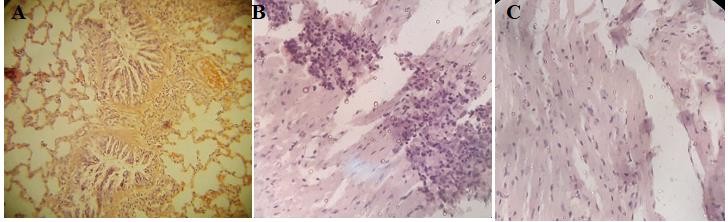
 S4 Fig. H& E section of guinea pigs lung (A) and heart (B;C) showing Profuse lymphocyte in filtration (400X magnification) from among the animals treated with 0.001mg/kg of TR.**

**S5 Fig. Serum Aspartate aminotransferase (AST or SGOT) (A), Alanine Aminotransferase (ALT or SGPT) (B), serum cholesterol (C), Serum Creatinine (D), blood Urea (E) at baseline and after 5th and 15th doses of TR @ 0.001mg/ kg administered by I/P route**

**300**


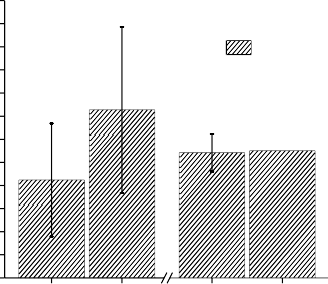


SGOT

**275**

**SGOT (IU/L)**

**A**

**250**

**225**

**200**

**175**

**150**

**125**

**100**

**75**

**50**

**25**

**0**

**0 5 15 20**

**Number of Transitmycin doses**

**B 75**

**70**


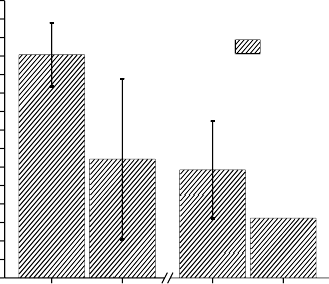


SGPT

**65**

**60**

**SGPT (IU/L)**

**55**

**50**

**45**

**40**

**35**

**30**

**25**

**20**

**15**

**10**

**5**

**0**

**0 5 15 20**

**Number of Transitmycin doses**

**C 45**

**Serum Cholesterol (mg/dL)**


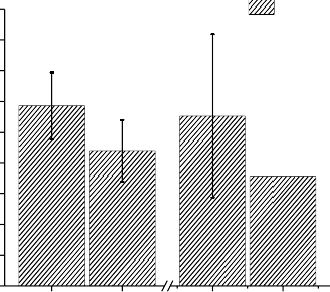


712

Cholesterol

**40**

**35**

**30**

**25**

**20**

**15**

**10**

**5**

**0**

**0 5 15 20**

**Number of Transitmycin doses**

**E**

**D**

**0.6**


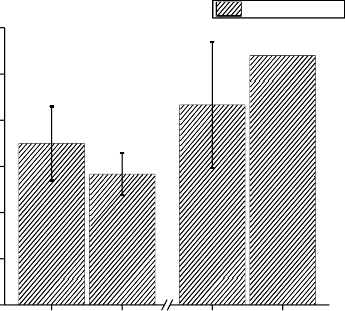


Serum Creatinine


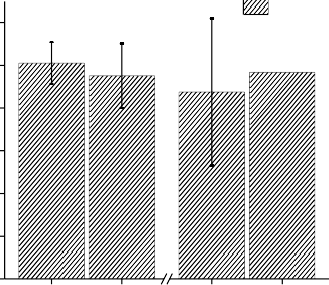


Blood urea

**60**

**Blood Urea (mg/dL)**

**0.5**

**50**

**Serum Creatinine (mg/dL)**

**0.4**

**40**

**30 0.3**

**20 0.2**

**10 0.1**

**0**

**0 5 15 20**

**0.0**

**Number of Transitmycin doses**

**0 5**

**Number of Transitmycin doses**

**S6A Fig. Viable bacterial numbers in whole lungs (A) and spleens (B) of *M. tuberculosis* infected guinea pigs treated with TR @ 0.001mg/kg (n=3 per group)**


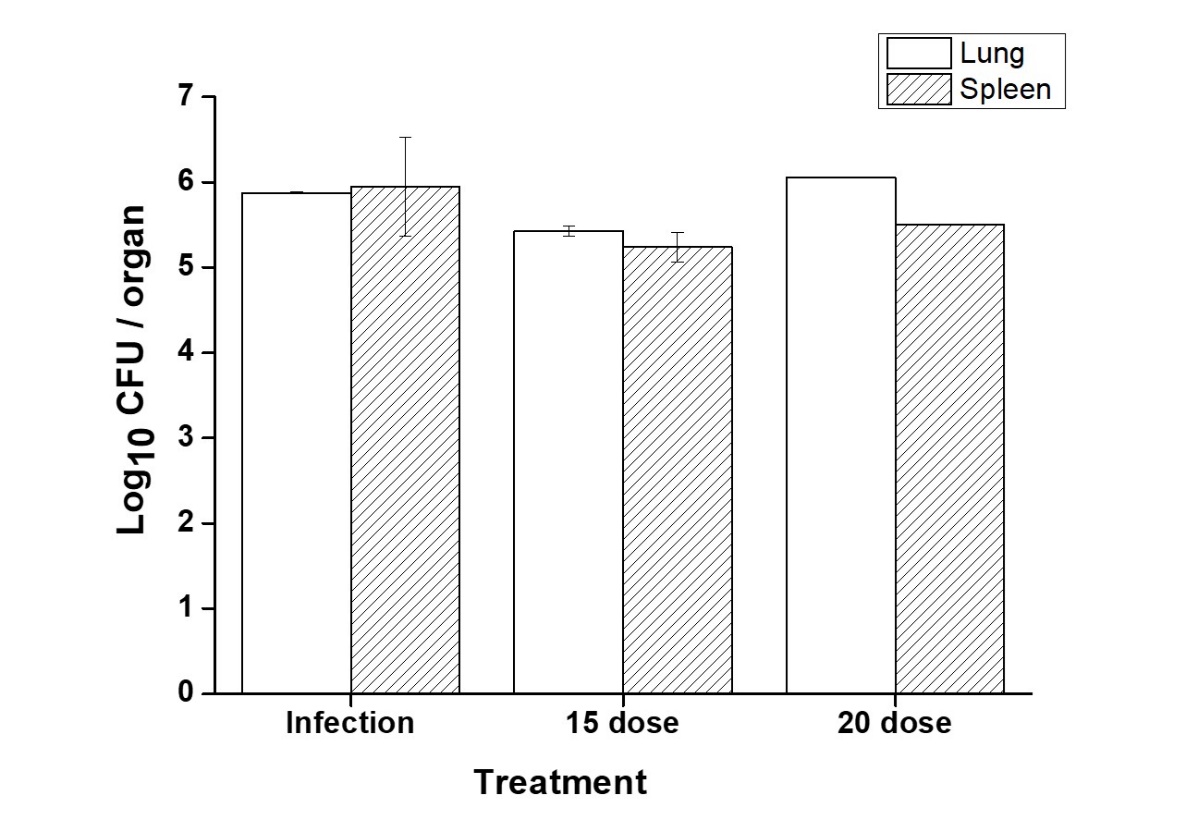


Antitubercular activity of the indicated drugs in infected guinea pigs. Animals were infected via aerosol route with ~100 CFU of M. tuberculosis H37Rv and were either left untreated or treated with drugs beginning 15 days after infection. The animals were treated with Transitmycin (Tra @ 0.001mg/kg every alternate day. Viable bacterial numbers in the whole lungs and spleens after 15th and 20th dose respectively. The results are expressed as the average (n=2) bacterial load in each group expressed as the log10 number of CFU (±SEM) per group at each time point except for guinea pigs treated with 20 doses of for which, n=1 per group.

**S6B Fig.** (A, B, C). The gross appearance of organs harvested from infected and untreated guinea pigs. (D, E, F) guinea pig infected with *M. tuberculosis H37Rv* and treated with TR drug @0.001mg/kg (total 15 doses) administered on alternate days via intraperitoneal route. (G, H, I): Guinea pig infected with *M. tuberculosis H37Rv* and treated with TR drug @0.001mg/kg (total 20 doses) administered on alternate days via intraperitoneal route.


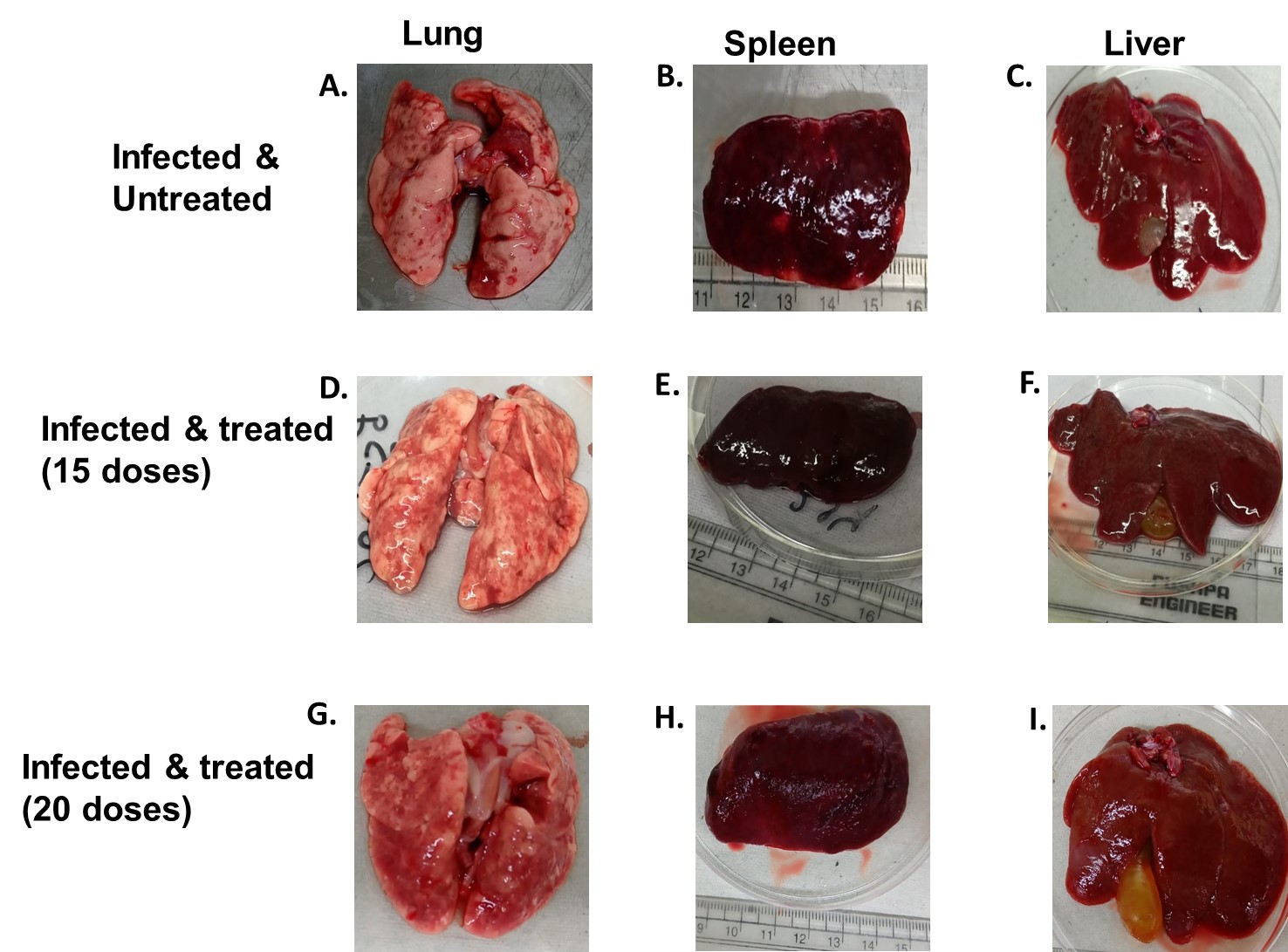


**S7 Fig. TR interaction with DNA (A) Interactions with the NH2 of Chromophore and GC Base Pair of DNA (B) Hydrogen Bonding and Pi-Pi bonding (C) 3D interaction with Peptide.**

| A | B | C |
| --- | --- | --- |


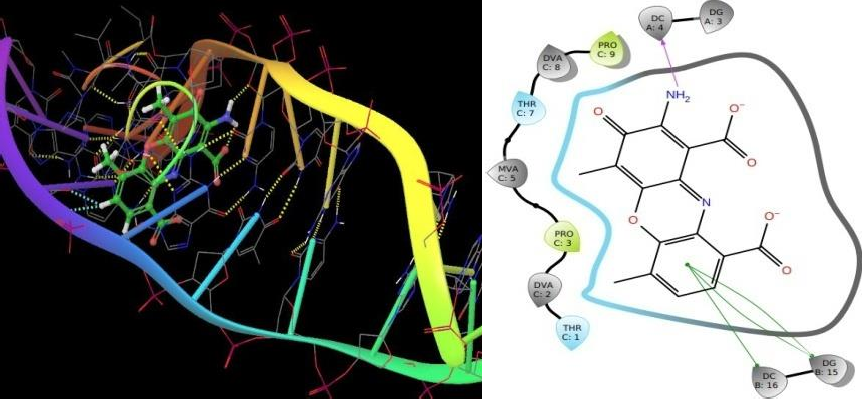

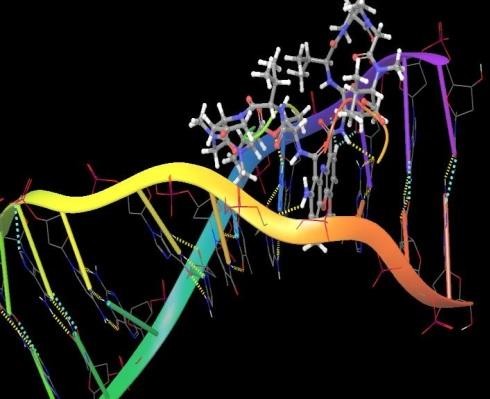


**S8 Fig. TR interaction with recombinase A (recA) (PDBID:1MO3) and Methionine Aminopeptidases (PDBID:3IU8 and 3PKE).The glide scores of TR interaction with 3IU8, 3PKE, and 1MO3 are -9.288, -4.774 and -7.689 respectively**.

| 3IU8withChromophore | 3PKEwith  Chromophore | 1MO3withChromophore |
| --- | --- | --- |
| 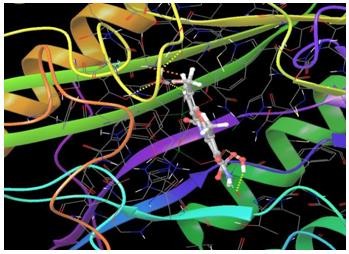 | 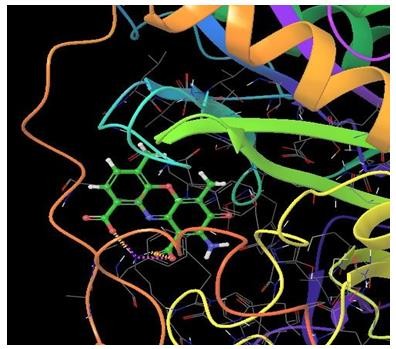 | 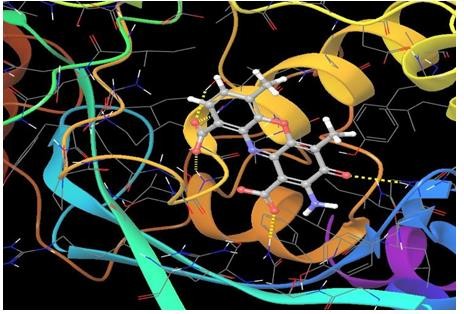 |

**S**
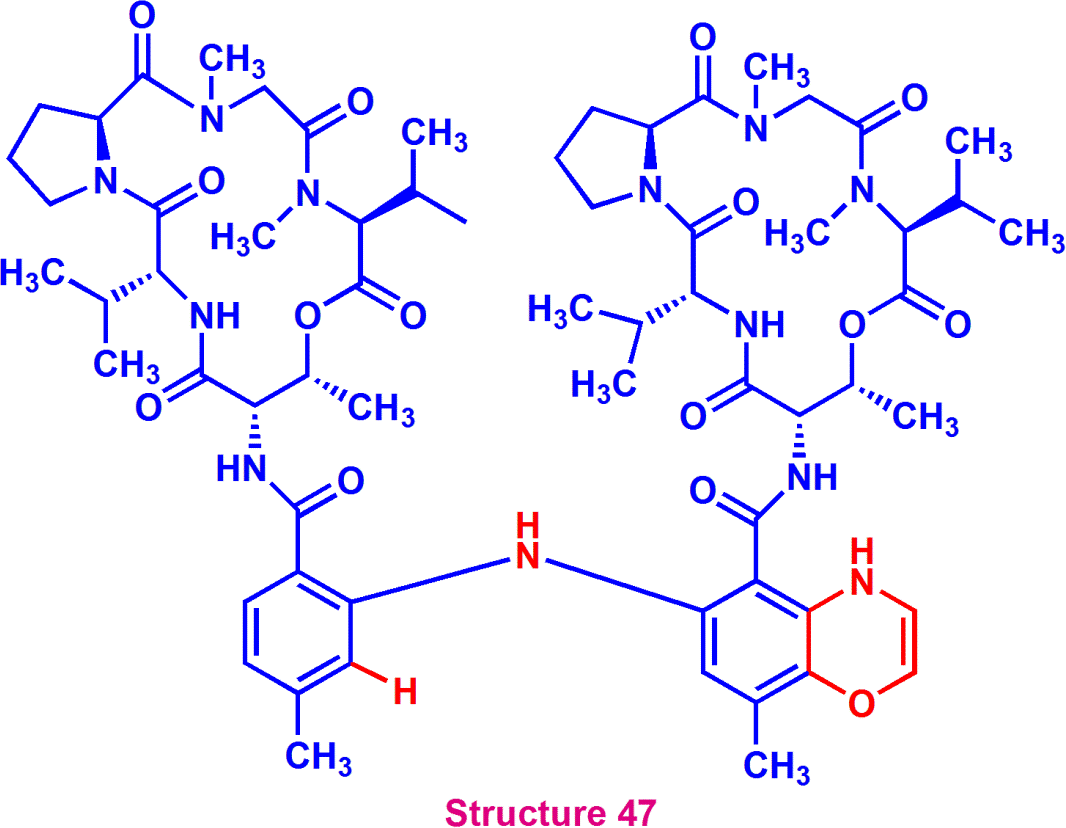
**9 Fig. Molecular Structure of TR analog compound 47**

**S10 Fig. In-vitro DNA intercalation tested by Ethidium Bromide displacement assay showing intercalation of DNA by TR and its analogs.**


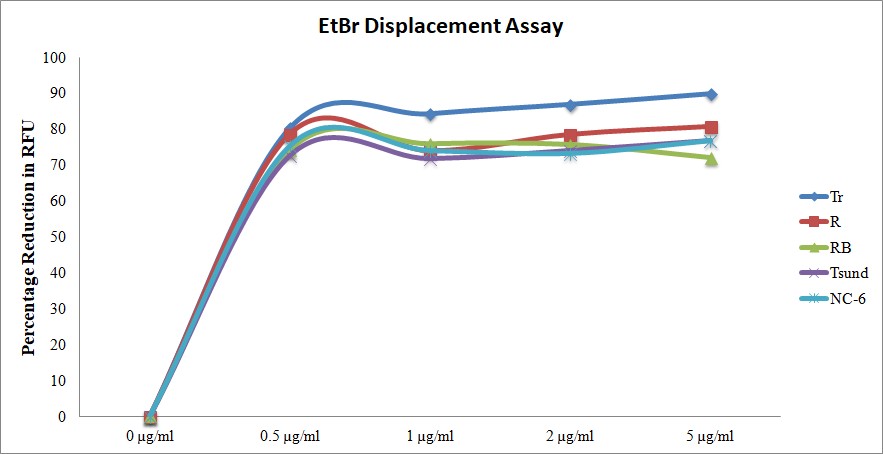


**S11 Fig. Viable bacterial numbers in whole lungs (A) and spleens (B) of *M. tuberculosis*-infected guinea pigs treated with INH+RIF, Erythromycin or TR @ 0.01mg kg^-1^, and 0.001 mg kg^-1^ respectively at 7^th^ , 12^th^ 15^th^ , 30^th^ and 40^th^ day post treatment corresponding to 3, 5, 7, 15 and 20 transitmycin doses respectively (n=2 per group at each time point)**

**S12 Fig. Effect of INH+RIF, Erythromycin or TR @0.01mg/kg, 0.02mg/kg, 0.04mg/kg respectively on kidney function parameter of guinea pigs at 7^th^, 12^th^ and 15^th^ days post-treatment: (A) Blood urea; (B) Serum creatinine**

**S13 Fig.** Liver morphology of guinea pigs treated: TR at 0.01mg/kg sacrificed after 3^rd^, 5^th^ and 6^th^ dose on day 7^th^, 12^th^ and 13^th^ post treatment (a, b & c) initiation respectively; TR@ 0.02mg/kg sacrificed after 3^rd^ dose on day 6^th^ and 7^th^ post treatment (d & e) initiation respectively; TR @ 0.04mg/kg sacrificed after 3^rd^dose on day 6^th^ and 7^th^ post treatment (f & g) initiation respectively.


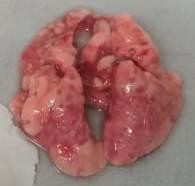

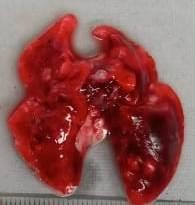

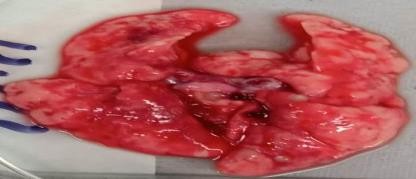
 a b c


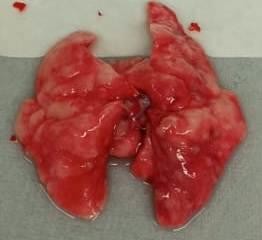


d


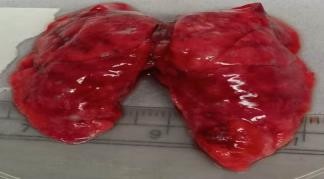


e

f


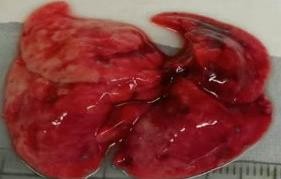


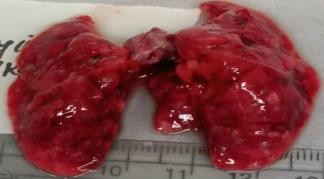


g

**S14.** **Fig. Synthesis of Transitmycin analogues**

1. **N-Alkylation of Transitmycin using 4-Hydroxy (hexyl)-Coumarin–[TR-NC6]**

Transitmycin (0.1 g, 0.078 mmol) was dissolved in 10 mL of N,N-Dimethyl formamide (DMF) solvent and was then added potassium carbonate (0.014 g, 0.10 mmole) stirred it room temperature for 30 minutes. Then was added 7-hexyl coumarin and mixture was stirred at 110 0C for 15 hours. Then after 15 hours the reaction quenched with addition of cold water. After separation of phases the aqueous phases was extracted with three times and the combined organic organic phases were dried using NaSO4, filtered, concentrated. Purification by neutral alumina column chromatography (5% Methanol:Ethyl acetate) gave 75% yield of N-alkylated transitmycin as reddish oranges powder.


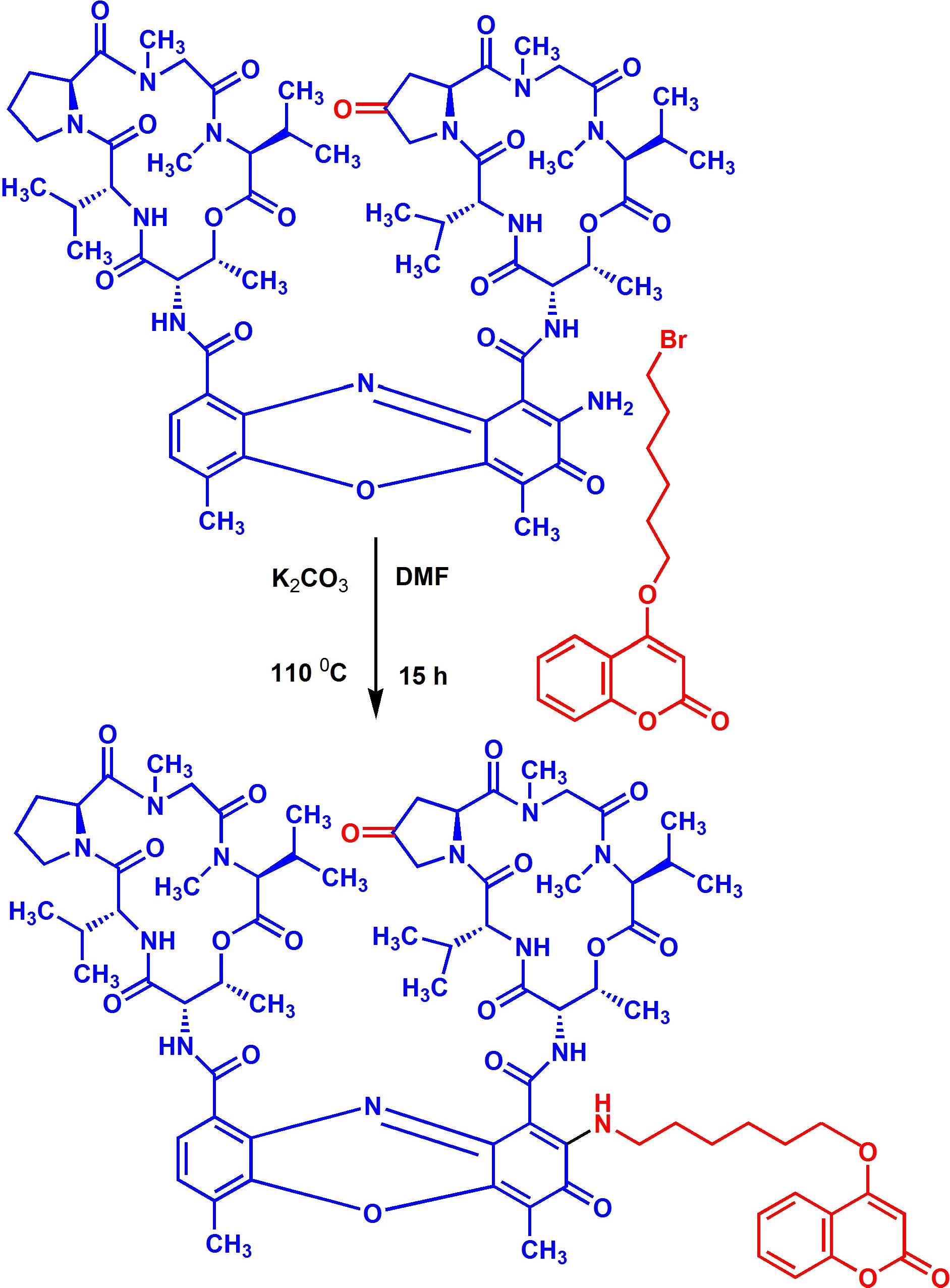


## TR Coumarin N-alkylation ^1^H NMR (500 MHZ, CDCl

| **S.No** | **TR** | **TRN** | **Coumarin** |
| --- | --- | --- | --- |
| **1** | 8.17 | 8.00 | 7.76 |
| **2** | 7.64 | 7.94 | 7.49 |
| **3** | 7.55 | 7.76 | 7.25 |
| **4** | 7.29 | 7.48 | 5.60 |
| **5** | 7.09 | 7.25 | 4.14 |
| **6** | 6.50 | 6.61 | 3.62 |
| **7** | 5.80 | 5.77 | 1.88 |
| **8** | 5.20 | 5.60 | 1.68 |
| **9** | 5.00 |  | 1.59 |
| **10** | 4.60 | 4.93 | 1.50 |
| **11** | 4.50 |  |  |
| **12** | 3.90 | 4.00 |  |
| **13** | 3.50 | 3.90 |  |
| **14** | 3.40 | 3.70 |  |
| **15** | 3.25 | 3.60 |  |
| **16** | 2.95 | 2.89  2.81 |  |
| **17** | 2.70 | 2.72 |  |
| **18** | 2.25 | 2.00 |  |
| **19** | 2.10 | 1.97 |  |
| **20** | 1.75 | 1.88 |  |
| **21** | 1.25 | 1.17-  1.21 |  |
| **22** | 1.15 |  |  |
| **23** | 0.90 |  |  |
| **24** | 0.60 |  |  |

**O**


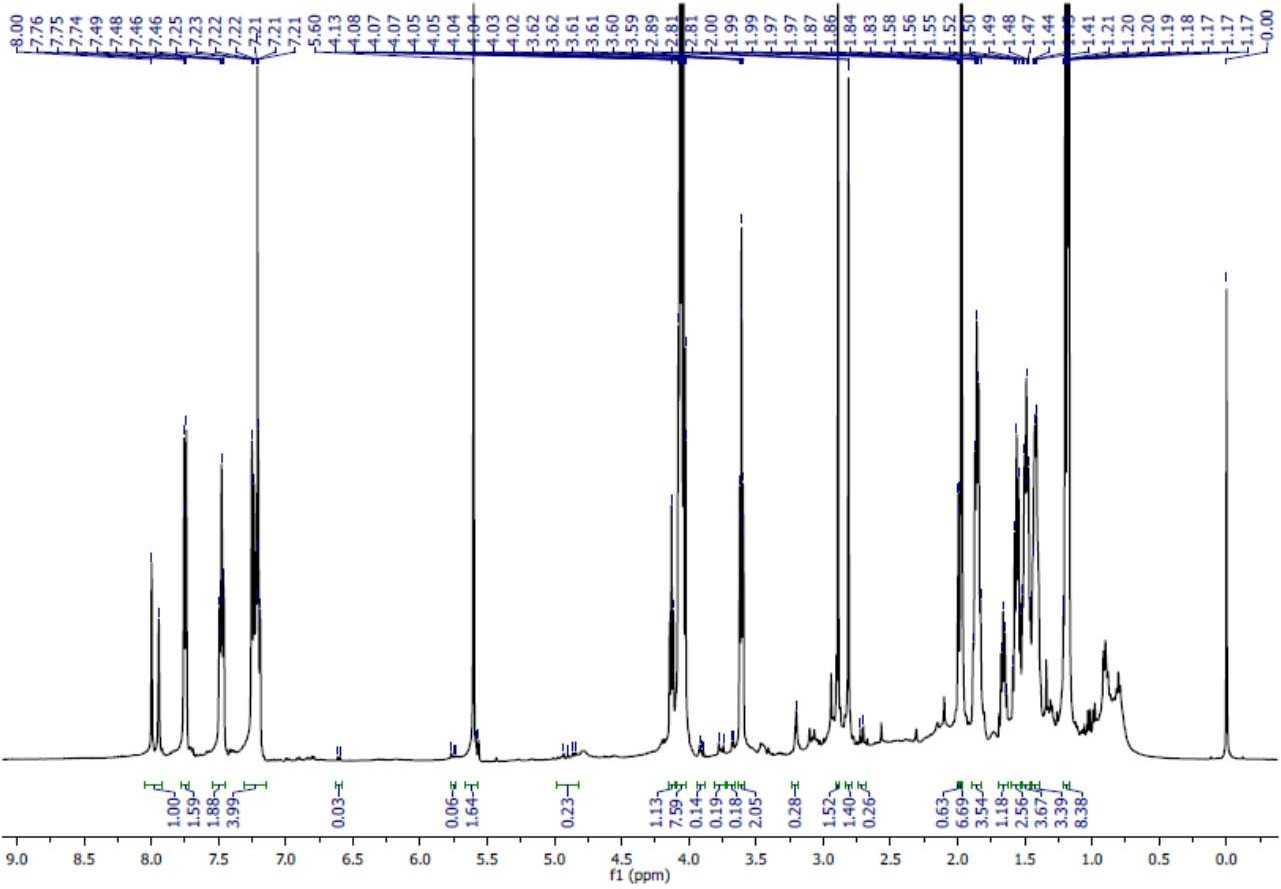


**^1^H NMR (500MHz, CDl3) δ** 8.00 (s,1H), 7.94 (s, 1H), 7.76 (d, *J*=10Hz, 2H), 7.49 (t, *J*=10Hz, 2H), 7.25-

7.19 (m,4H), 6.61 (d, *J*=5Hz, 1H), 5.77-5.73 (m), 5.60-5.57 (m), 4.93-4.84 (m), 4.14 (t, *J*=5Hz, 2H), 4.08-

4.02 (M), 3.92-3.89 (m), 3.78 (s), 3.74 (s), 3.68 (d, *J*=5Hz,1H), 3.62 (t, *J*=5Hz, 2H), 3.20 (s), 2.89 (s), 2.81

(s), 2.72 (d, *J*=10Hz, 1H), 2.00 (d, *J*=5Hz, 1H), 1.97 (s), 1.88-1.83 (m, 2H), 1,68-1.63 (m, 2H), 1.59-1.53

(m, 2H), 1.52-1.49 (m, 2H), 1.48-1.41 (m, 2H), 1.21-1.17 (m) ppm. **Chemical Formula**: C77H100N12O20;

**Exact Mass (m/z)**: 1512.7177.

**O**

**N**

**N O**

**O O**

**O**

**O**

**O**

**O**

**O**

**N**

**O**

**N**

**O**

**N**

**N**

**N H**

**O**

**HN**

**H N**

**O**

**O**

**H N**

**O**

**O**

**NH O O**

**N**

**O**

## N-Glycosylation of Transitmycin using Acetobromo-α-D-Glucose [Tr-SUND]

- 1. Transitmycin (0.1 g, 0.078 mmol) was dissolved in 10 mL N-N-Dimethyl formamide (DMF) solvent and was then added cesium carbonate (0.032 g, 0.014) stirred it room temperature for 30 minutes. Then was added 1-bromo-α-D-glucose tetra acetate (0.0057 g,

0.014 mmole) and mixture was stirred at 110 0C for 15 hours. Then after 15 hours the reaction quenched with addition of cold water. After separation of phases the aqueous phases was extracted with three times and the combined organic organic phases were dried using NaSO4, filtered, concentrated. Purification by neutral alumina column chromatography (5% Methanol:Ethyl acetate) gave N-glycosylated tetra acetate transitmycin as reddish oranges liquid.

- 1. N-glycosylated tetra acetate Transitmycin (0.1 g, 0.078 mmol) was dissolved in methanol and cooled to 0 0C. Then was added 5 small pieces of sodium metal and the mixture was stirred at 0 0C for 1 h and directly transferred to column chromatography without workup. Purification by neutral alumina column chromatography (50% Methanol:Ethyl acetate) gave N-glcosylated transitmycin (Yield 50%) as reddish liquid.


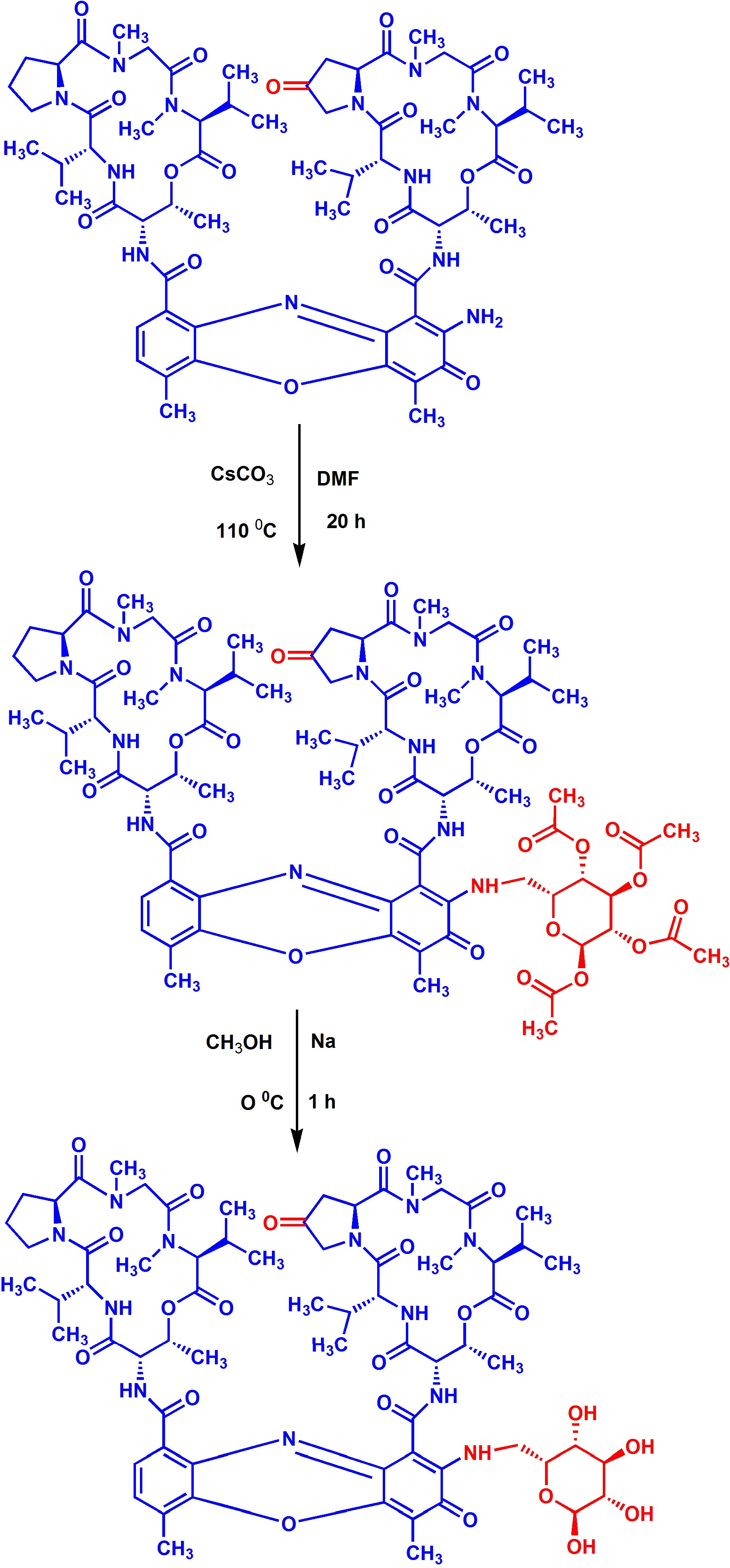
Note: The product was highly water soluble.

**TRSu N ^1^H NMR (500MHZ, CDCl3)**

| **S.No** | **TR** | **TRSuND** | **Sugar** |
| --- | --- | --- | --- |
| **1** | 8.17 | 8.58 | 6.54 |
| **2** | 7.64 | 7.75 | 6.04 |
| **3** | 7.55 | 7.56 | 5.16 |
| **4** | 7.29 | 7.36 | 4.34 |
| **5** | 7.09 | 7.28 | 2.54 |
| **6** | 6.50 | 6.77 |  |
| **7** | 5.80 | 5.87 |  |
| **8** | 5.20 | 5.48 |  |
| **9** | 5.00 | 5.02 |  |
| **10** | 4.60 | 4.45 |  |
| **11** | 4.50 |  |  |
| **12** | 3.90 | 3.78 |  |
| **13** | 3.50 | 3.59 |  |
| **14** | 3.40 | 3.47 |  |
| **15** | 3.25 | 3.18 |  |
| **16** | 2.95 | 2.67-  2.24 |  |
| **17** | 2.70 |  |  |
| **18** | 2.25 |  |  |
| **19** | 2.10 | 1.84 |  |
| **20** | 1.75 | 1.70-  1.52 |  |
| **21** | 1.25 |  |  |
| **22** | 1.15 | 1.20 |  |
| **23** | 0.90 | 0.92-  0.49 |  |
| **24** | 0.60 |  |  |


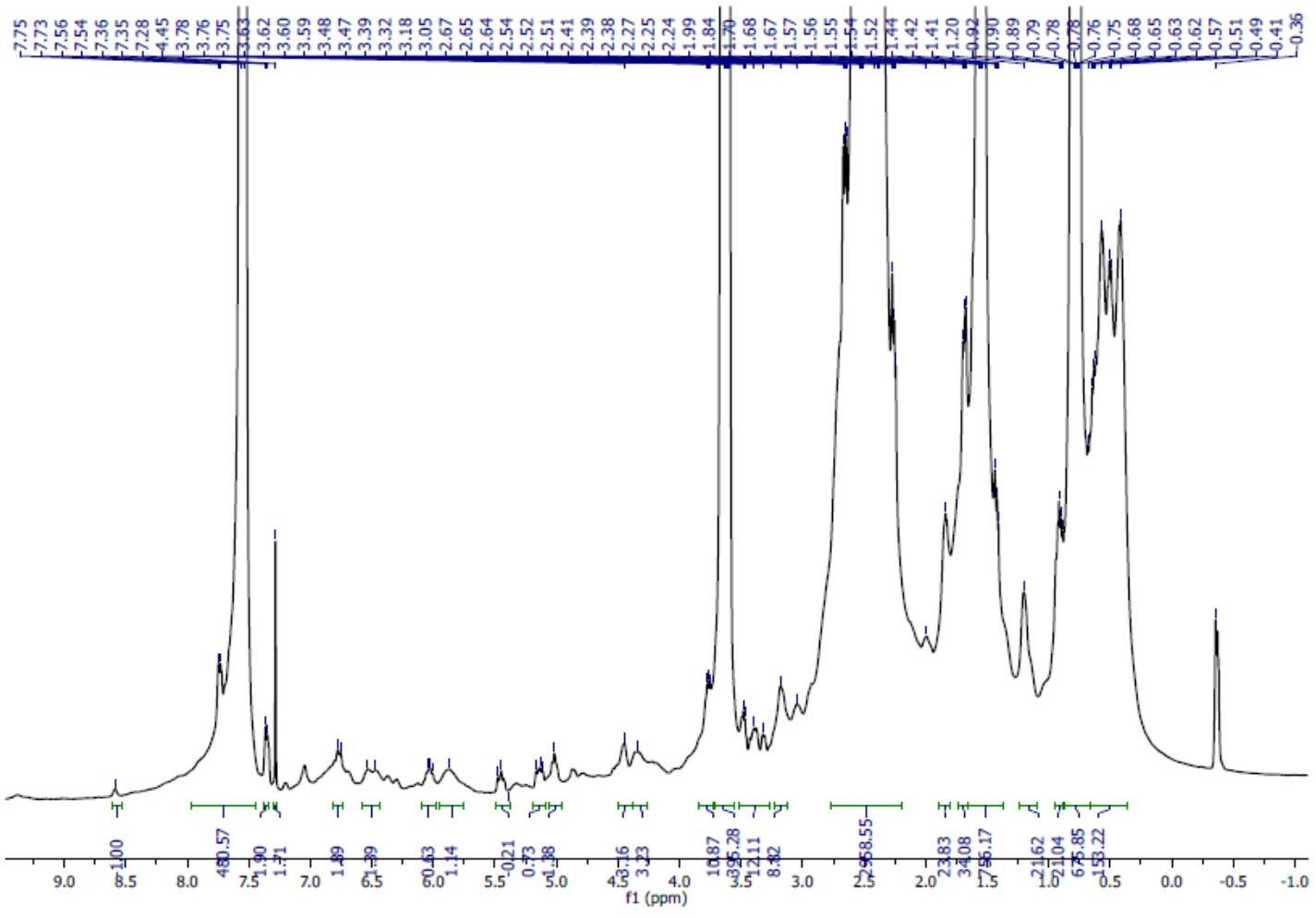


***6-(1-((6R,9R,10S,13S,18aR)-6,13-diisopropyl-2,5,9-trimethyl-1,4,7,11,14,17-hexaoxohexadecahydro- 1H-pyrrolo[2,1-i][1,4,7,10,13]oxatetraazacyclohexadecin-10-ylcarbamoyl)-9-((6S,9R,10R,13S,18aR)- 6,13-diisopropyl-2,5,9-trimethyl-1,4,7,11,14-pentaoxohexadecahydro-1H-pyrrolo[2,1- i][1,4,7,10,13]oxatetraazacyclohexadecin-10-ylcarbamoyl)-4,6-dimethyl-3-oxo-3H-phenoxazin-2- ylamino)tetrahydro-2H-pyran-2,3,4,5-tetrayl tetraacetate.***

**^1^H NMR (500MHz, CDl3) δ** 8.58 (s), 7.75 (d, J=10Hz), 7.56 (d, J=10Hz), 7.36 (d, J=5Hz), 7.28 (s), 6.77

(d, J=10Hz), 6.54 (d,J=20Hz), 6.04 (t, J=5Hz), 5.87 (s), 5.48 (s), 5.16 (t, J=10Hz), 5.02 (s), 4.45 (s), 4.34

(s), 3.78 (s), 3.59 (s), 3.47 (s), 3.18 (s)2.67-2.24 (m), 1.84 (s), 1.70-1.52 (m), 1.20 (s), 0.92-0.49(m) ppm.

**Chemical Formula**: C75H100N12O26; **Exact Mass (m/z)**: 1584.6872


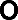

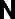

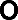

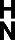

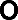

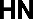

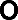

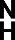

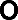

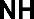

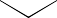

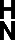

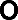

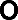

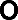

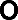

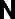

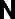

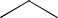

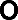

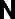

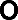

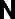

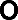

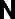

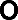

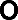

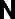

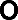

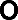

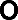

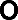

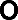

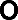

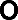

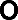

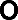

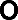

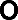

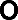


**TRSuN ^1^H NMR (500MHZ, CDCl3)**

| **S.No** | **TR** | **TRSuND** | **Sugar** |
| --- | --- | --- | --- |
| **1** | 8.17 | 8.58 | 6.54 |
| **2** | 7.64 | 7.75 | 6.04 |
| **3** | 7.55 | 7.56 | 5.16 |
| **4** | 7.29 | 7.36 | 4.34 |
| **5** | 7.09 | 7.28 | 2.54 |
| **6** | 6.50 | 6.77 |  |
| **7** | 5.80 | 5.87 |  |
| **8** | 5.20 | 5.48 |  |
| **9** | 5.00 | 5.02 |  |
| **10** | 4.60 | 4.45 |  |
| **11** | 4.50 |  |  |
| **12** | 3.90 | 3.78 |  |
| **13** | 3.50 | 3.59 |  |
| **14** | 3.40 | 3.47 |  |
| **15** | 3.25 | 3.18 |  |
| **16** | 2.95 | 2.67-  2.24 |  |
| **17** | 2.70 |  |  |
| **18** | 2.25 |  |  |
| **19** | 2.10 | 1.84 |  |
| **20** | 1.75 | 1.70-  1.52 |  |
| **21** | 1.25 |  |  |
| **22** | 1.15 | 1.20 |  |
| **23** | 0.90 | 0.92-  0.49 |  |
| **24** | 0.60 |  |  |


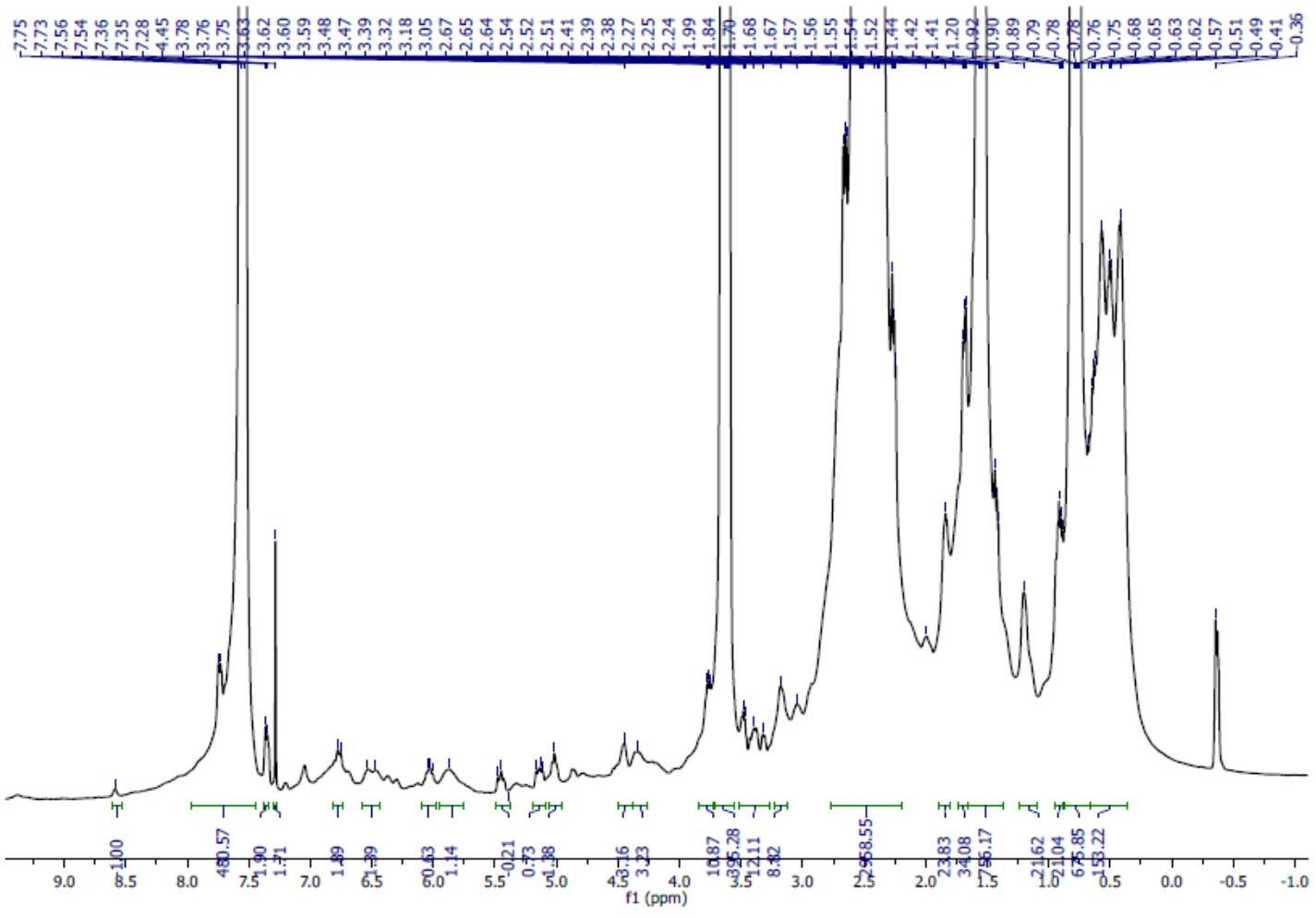


***6-(1-((6R,9R,10S,13S,18aR)-6,13-diisopropyl-2,5,9-trimethyl-1,4,7,11,14,17-hexaoxohexadecahydro- 1H-pyrrolo[2,1-i][1,4,7,10,13]oxatetraazacyclohexadecin-10-ylcarbamoyl)-9-((6S,9R,10R,13S,18aR)- 6,13-diisopropyl-2,5,9-trimethyl-1,4,7,11,14-pentaoxohexadecahydro-1H-pyrrolo[2,1- i][1,4,7,10,13]oxatetraazacyclohexadecin-10-ylcarbamoyl)-4,6-dimethyl-3-oxo-3H-phenoxazin-2- ylamino)tetrahydro-2H-pyran-2,3,4,5-tetrayl tetraacetate.***

**^1^H NMR (500MHz, CDl3) δ** 8.58 (s), 7.75 (d, J=10Hz), 7.56 (d, J=10Hz), 7.36 (d, J=5Hz), 7.28 (s), 6.77

(d, J=10Hz), 6.54 (d,J=20Hz), 6.04 (t, J=5Hz), 5.87 (s), 5.48 (s), 5.16 (t, J=10Hz), 5.02 (s), 4.45 (s), 4.34

(s), 3.78 (s), 3.59 (s), 3.47 (s), 3.18 (s)2.67-2.24 (m), 1.84 (s), 1.70-1.52 (m), 1.20 (s), 0.92-0.49(m) ppm.

**Chemical Formula**: C75H100N12O26; **Exact Mass (m/z)**: 1584.6872

## Reduction of keto group in the Transitmycin using sodium borohydride (NaBH4)-[TR-R]

To a stirred solution of Transitmycin (0.1 g, 0.078 mmole) in 10 mL of methanol was successively added sodium borohydride (NaBH4) (0.156 mmole, 0.006 g). The reaction mixture was stirred for 60 min at room temperature reaction after 60 minutes the reaction was quenched with addition of 1 mL of water and 2-3 drops of dil. HCl, then evaporate the methanol under rotary evaporator then added less amount of water followed by ethyl acetate and phases were separated. The aqueous phasewere extracted with ethyl acetate three times, and the combined organic phases were using Na2SO4, filtered and concentrated. Purification using neutral alumina column chromatography (10% Methanol:Ethylacetate) yields (50%) of TR-R as pale yellow solid.

Note: The product was highly water soluble.


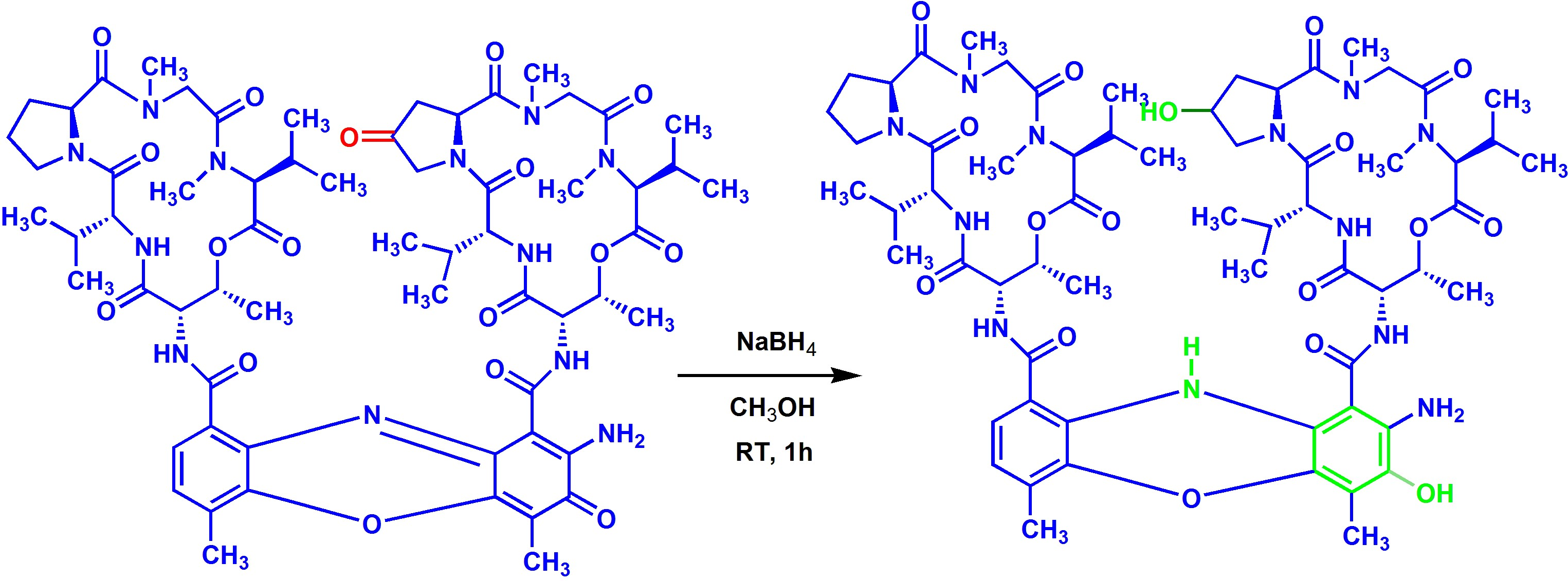


## Benzoylation of Transitmycin-OH using 4-Methoxy-Benzoyl Chloride –[TR-RB]

To a stirred solution of 10% aqueous sodium hydroxide (NaOH) was added Transitmycin- OH (0.1 g, 0.0786 mmole ) the mixture was stirred for 30 minutes at room temperature, after 30 minutes was added p-methoxy benzyl chloride (0.012 g, 0.0094 mmole) continue the stirring at room temperature for another 1 hour, after which mixture was acidified with dilute HCl until pH= 3, the aqueous layer was extracted ethyl acetate with three times, and the combined organic layers were dried (Na2SO4), filtered and concentrated. Purification by neutral alumina column chromatography (10% Methanol: Ethylacetate) gave TR-RB (Yield 80%) as pale yellow solid.


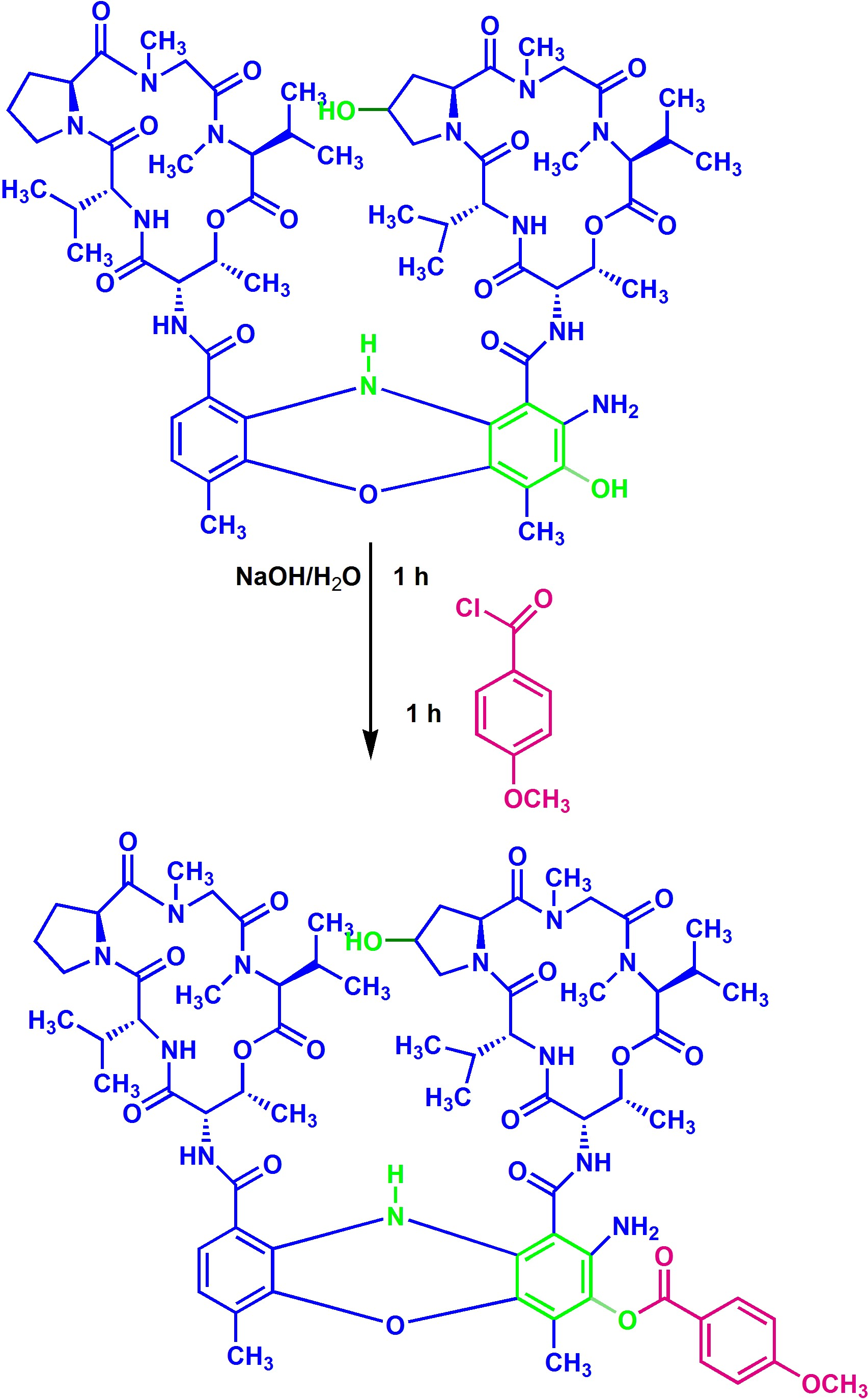


**TR RB ^1^H NMR (500MHZ, CDCl3)**

| **S.No** | **TR** | **TR-RB** | **Benzoyl** |
| --- | --- | --- | --- |
| **1** | 8.17 | 8.14 | 7.98 |
| **2** | 7.64 | 7.98 | 7.19 |
| **3** | 7.55 | 7.80 | 6.87 |
| **4** | 7.29 | 7.47 |  |
| **5** | 7.09 | 6.68 |  |
| **6** | 6.50 | 6.55 |  |
| **7** | 5.80 | 5.75 |  |
| **8** | 5.20 | 5.23 |  |

| **9** | 5.00 |  |  |
| --- | --- | --- | --- |
| **10** | 4.60 | 4.94 |  |
| **11** | 4.50 |  |  |
| **12** | 3.90 | 4.06 |  |
| **13** | 3.50 | 3.94 |  |
| **14** | 3.40 | 3.80 |  |
| **15** | 3.25 | 3.65 |  |
| **16** | 2.95 | 2.90 |  |
| **17** | 2.70 | 2.59 |  |
| **18** | 2.25 | 2.20 |  |
| **19** | 2.10 | 2.03 |  |
| **20** | 1.75 | 1.98 |  |
| **21** | 1.25 | 1.36-  0.76 |  |
| **22** | 1.15 |  |  |
| **23** | 0.90 |  |  |
| **24** | 0.60 |  |  |


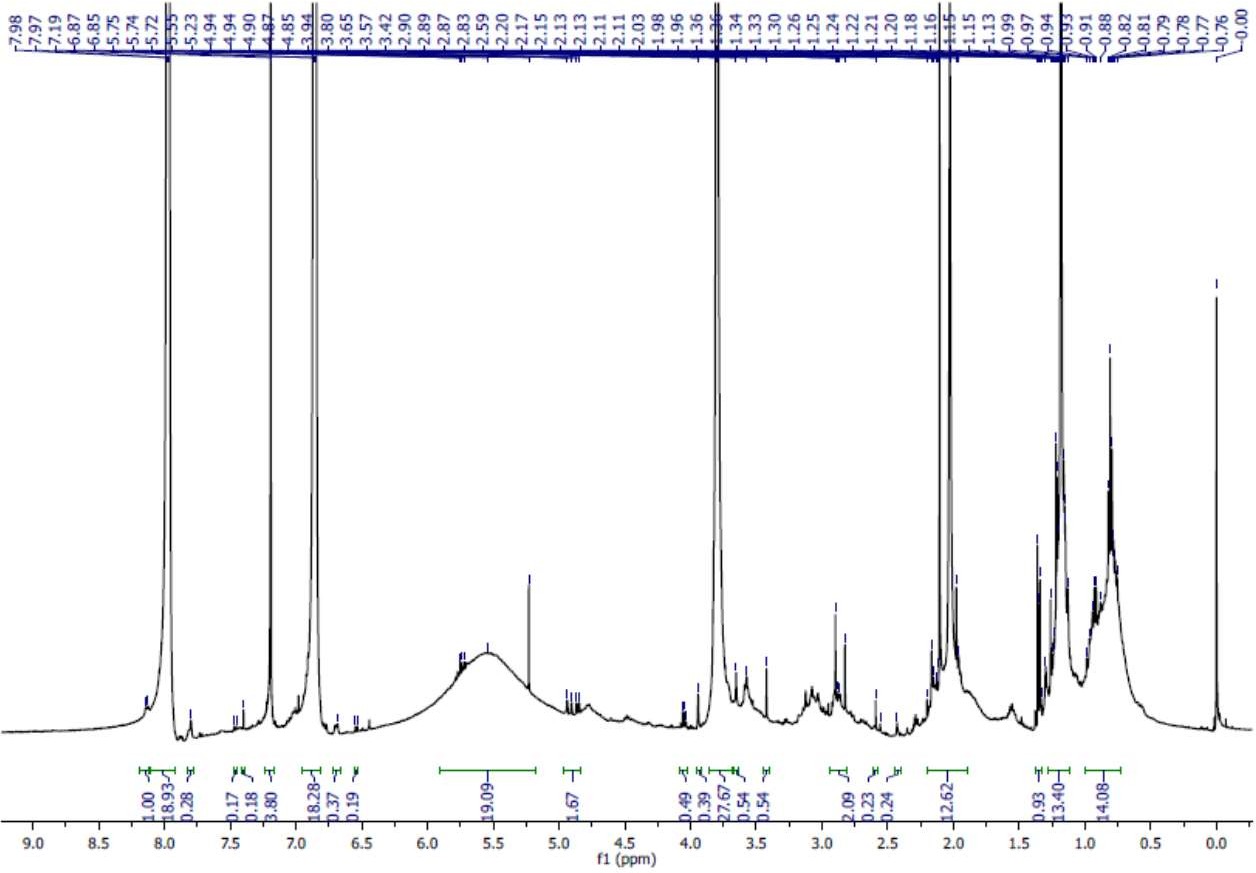


***2-amino-9-((6S,9R,10R,13S,18aR)-6,13-diisopropyl-2,5,9-trimethyl-1,4,7,11,14 pentaoxohexadecahydro-1H-pyrrolo[2,1-i][1,4,7,10,13]oxatetraazacyclohexadecin-10-ylcarbamoyl)-1- ((6R,9R,10S,13S,18aR)-17-hydroxy-6,13-diisopropyl-2,5,9-trimethyl-1,4,7,11,14- pentaoxohexadecahydro-1H-pyrrolo[2,1-i][1,4,7,10,13]oxatetraazacyclohexadecin-10-ylcarbamoyl)- 4,6-dimethyl-10H-phenoxazin-3-yl benzoate*.**

**^1^H NMR (500MHz, CDl3) δ** 8.14 (d, *J*=5Hz), 7.98 (d, *J*=5Hz), 7.80 (s), 7.47 (d, *J*=10Hz), 7.19 (s), 6.87

(d, *J*=10Hz), 6.68 (d, *J*=10Hz), 6.55 (d, *J*=10Hz), 5.75-5.72 (m), 5.55 (bs), 5.23 (s), 4.94-4.85 (m), 4.06-

4.03 (m), 3.94(s), 3.80 (s), 3.65 (s), 2.90 (-2.83), 2.59 (s), 2.20 (s), 2.03 (s), 1.98 (s), 1.36-0.76 (m) ppm.

**Chemical Formula**: C69H92N12O18; **Exact Mass (m/z)**: 1376.6653.

**O**

**O**

**HO**

**N**

**N N**

**O**

**NH O O**

**O**

**O**

**H N**

**O**

**O**

**HN O**

**N H**

**N**

**H2N**

**H N**

**O**

**N O**

**N**

**O**

**O**

**O**

**O**

**O**

# Code: FN-Me


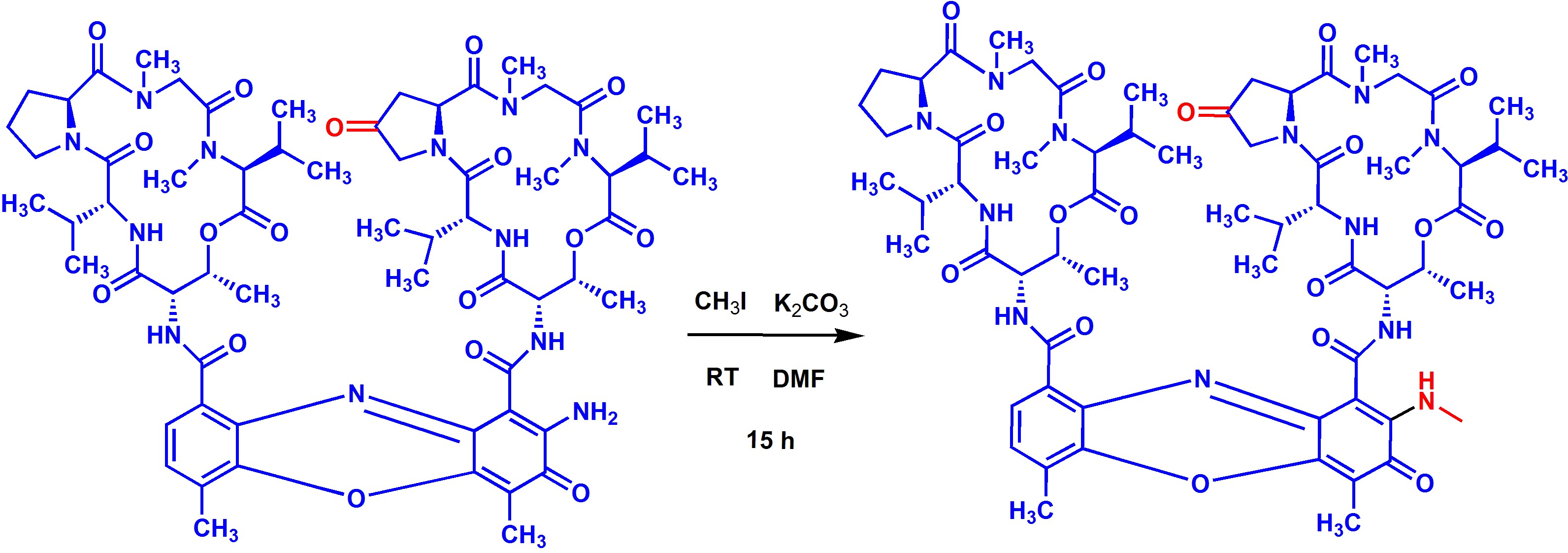


## 1. N-Alkylation of Transitmycin using Iodomethane [FN-Me]

Transitmycin (0.1 g, 0.078 mmol) was dissolved in 10 mL of N,N-Dimethyl formamide (DMF) solvent and was then added potassium carbonate (0.0217 g, 0.156 mmole) stirred it room temperature for 30 minutes. Then was added iodomethane (20 μl, 0.312 mmole) and mixture was stirred at room temperature for 15 hours. Then after 15 hours the reaction quenched with addition of cold water. After separation of phases the aqueous phases was extracted with three times and the combined organic organic phases were dried using NaSO4, filtered, concentrated. Purification by neutral alumina column chromatography (5% Methanol:Ethyl acetate) gave 80% yield of N- methylated transitmycin as reddish oranges powder.

## ^1^ H NMR (500 MHz, CDCl3) Spectrum of N-Methylated of Transitmycin using Iodomethane [FN-Me]


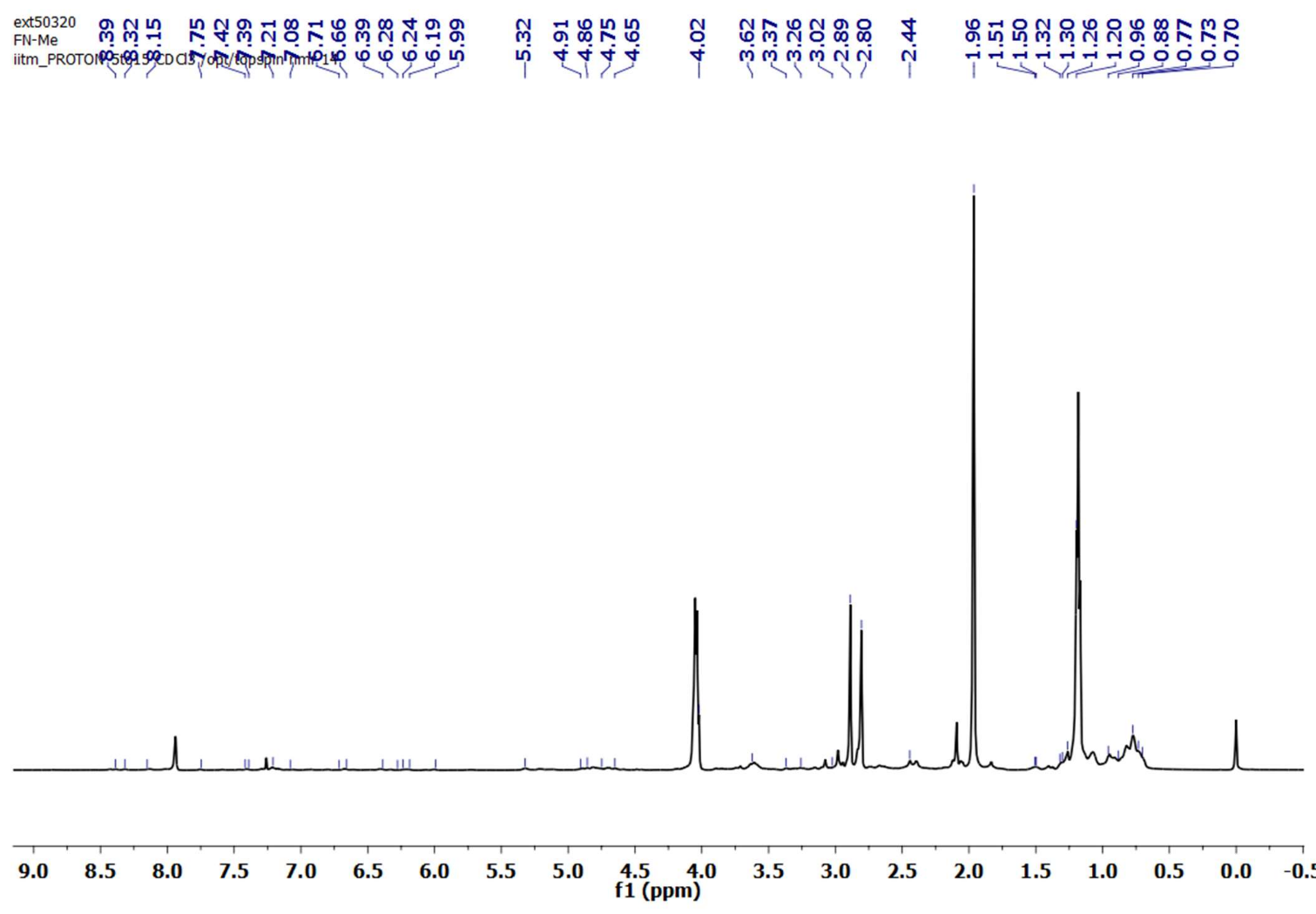

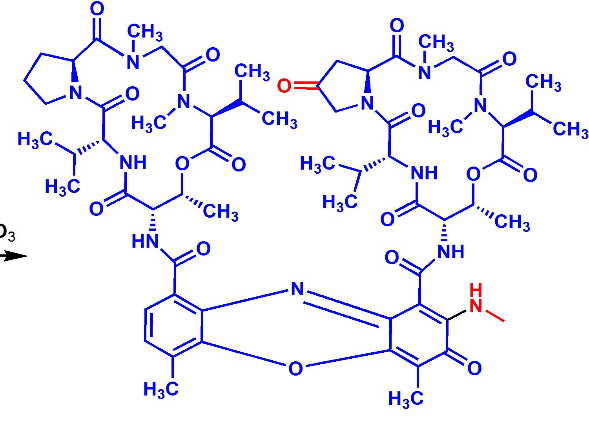


**Code: TR-Tert butyl**


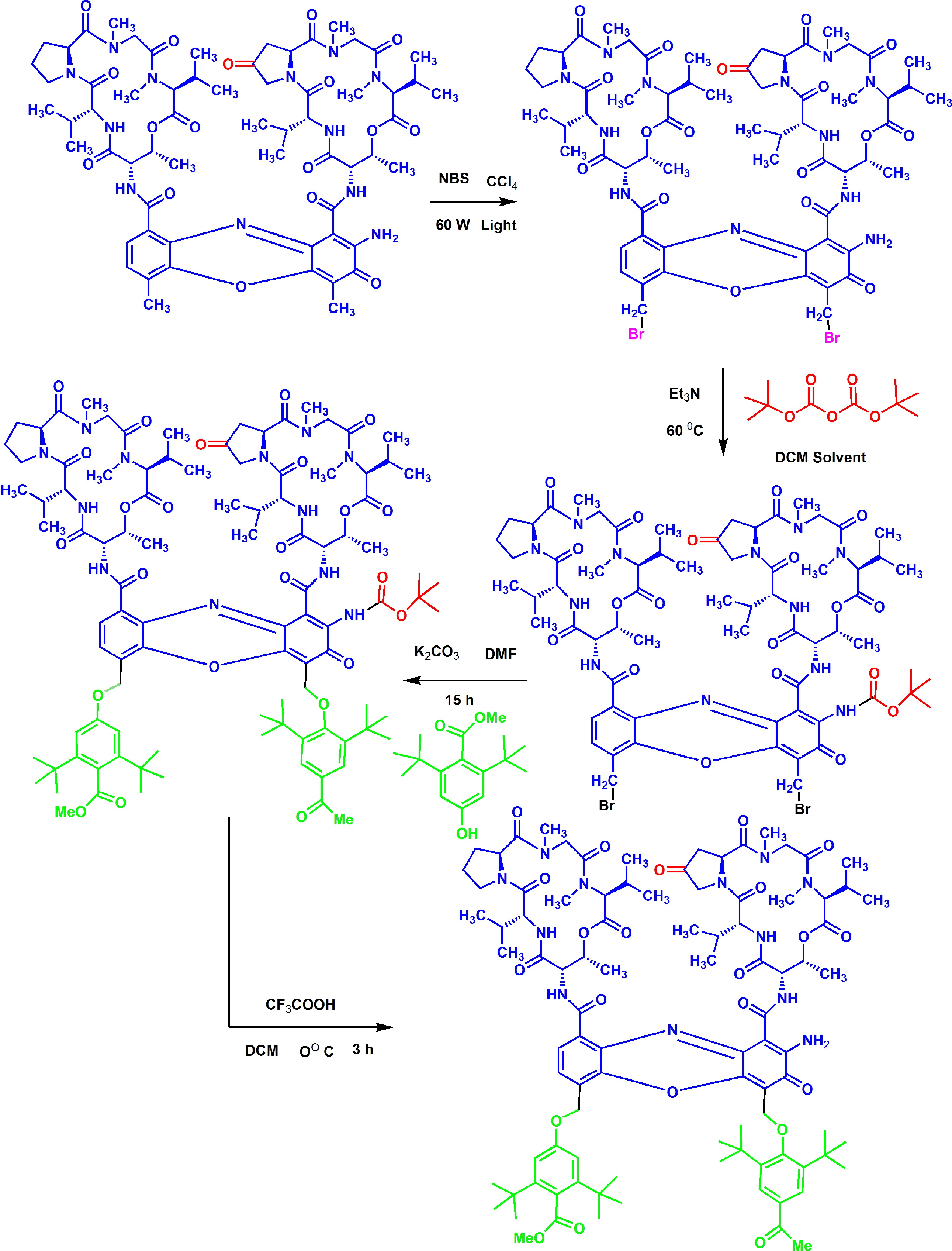


**Step 1: Bromination at Methyl group of chromophore of Transitmycin**

Transitmycin (0.078 mmole, 0.1 g) and an equipmolar amount of *N*-bromosuccinamide (0.078 mmole, 0.0139 g) which were dissolved in 20 mL of CCl4 and mixture was stirred at room temperature vigorously. Then was added catalytic amount of radical initiators dibenzoyl peroxide (DBP) in to the reaction mixture and mixture as illuminated with a 40 W incandescent light bulb for 5 hours. The reaction was allowed to cool to room temperature and a mixture of saturated aq. NH4Cl (10 mL) and water (10 mL) was added. The organic phase was separated, washed with brine (30 mL), dried (Na2SO4) and concentrated under reduced pressure to yield crude bromo product which was purified by neutral alumina column chromatography using (80% EtOAc/Hexane) to give bromo compound as a red colour powder.

## Step 2: BOC protection of amine group prevsent in chromophore of Transitmycin

To a stirred solution of Transitmycin (0.1 g , 0.078 mmole,) Boc anhydride (0.034g, 0.156 mmole) in CH2Cl2 (20 mL) at 0 °C and the NEt3 (0.0238 ml, 0.1716 mmol,) was added slowly. The mixture was stirred at reflux condition for 15 h. A mixture of saturated aq. NH4Cl (15 mL) and water (10 mL) was added, the organic phase was separated and the aqueous phase was back-extracted with CH2Cl2 (2 × 20 mL). The combined organics were washed with a mixture of saturated aq. NH4Cl (5 mL) and water (10 mL), saturated aq. NaHCO3 (5 mL), brine (5 mL), dried (Na2SO4) and concentrated under reduced pressure. The residue was purified by neutral alumina column chromatography (EtOAc/hexane) to afford the corresponding BOC protected bromo Transimycin as product.

## Step 3: O-alkylation of Transitmycin

Dibromo transitmycin (0.1 g, 0.078 mmol) was dissolved in 10 mL of N,N-Dimethyl formamide (DMF) solvent and was then added potassium carbonate (0.0215 g, 0.156 mmole,) stirred it room temperature room for 30 minutes. Then was added 3,5-Ditert-buty-4-hydroxy benzoic acid methyl ester (0.041 g , 0.156 mmole) and mixture was stirred it 110 0C for 15 hours. Then after 15 hours the reaction quenched with addition of cold water. After separation of phases the aqueous phases was extracted with three times and the combined organic phases were dried using NaSO4, filtered, concentrated. Purification by neutral alumina column chromatography (5%

Methanol:Ethyl acetate) gave 80% yield of BOC protected O-alkylated transitmycin as reddish oranges powder.

## Step 4: BOC-deprotection

To a cold (0°C) solution of above compound (0.1 g, 0.078 mmol) in DCM (10 mL) was added trifluoroacetic acid (TFA), (100 μl) and the mixture was stirred at 0 °C for 3 h. A mixture of saturated aq. NH4Cl (5 mL) and water (5 mL) was added, the organic phase was separated and the aqueous phase was back-extracted with CH2Cl2 (2 × 10 mL). The combined organics were washed with a mixture of saturated aq. NH4Cl (5 mL) and water (5 mL), saturated aq. NaHCO3 (5 mL), brine (5 mL), dried (Na2SO4) and concentrated under reduced pressure to obtain the desired product as red colour solid. The residue was purified by neutral alumina column chromatography (EtOAc/ hexane) to afford the corresponding pure product.

## Step 1: Bromination at Methyl group of chromophore of Transitmycin


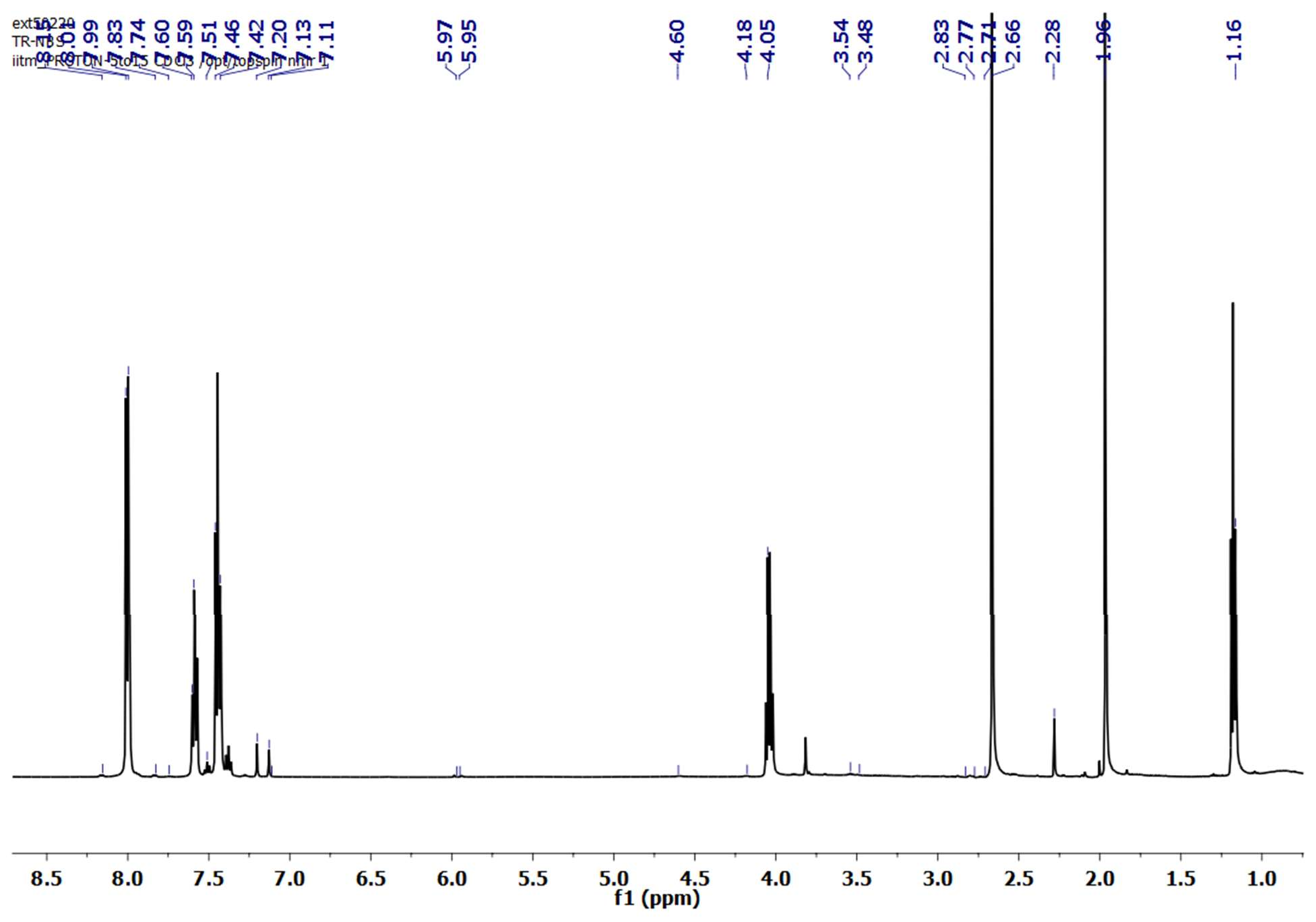


**Step 2: BOC protection of amine group prevsent in chromophore of Transitmycin**


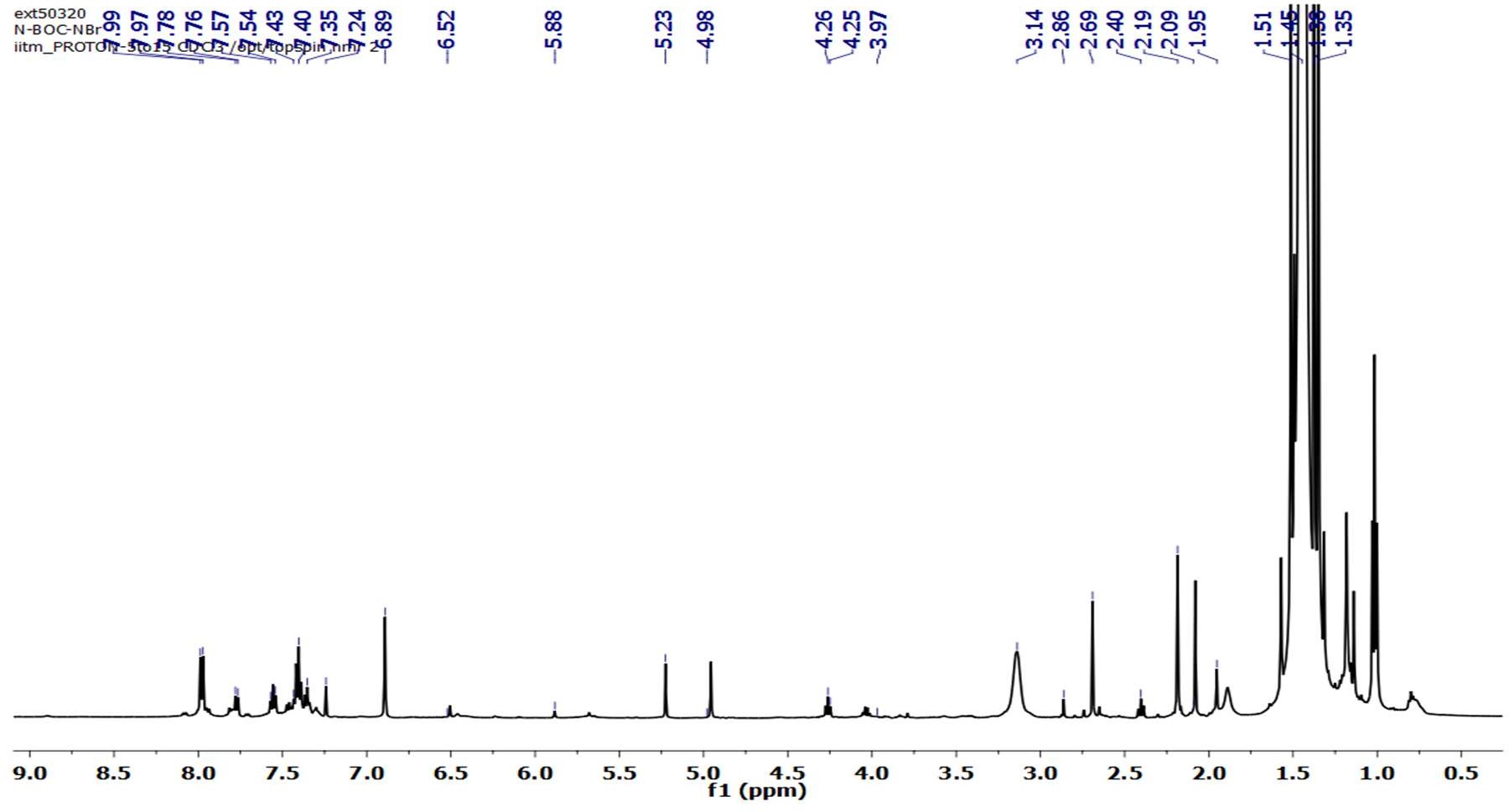


**Step 3: O-alkylation of Transitmycin**


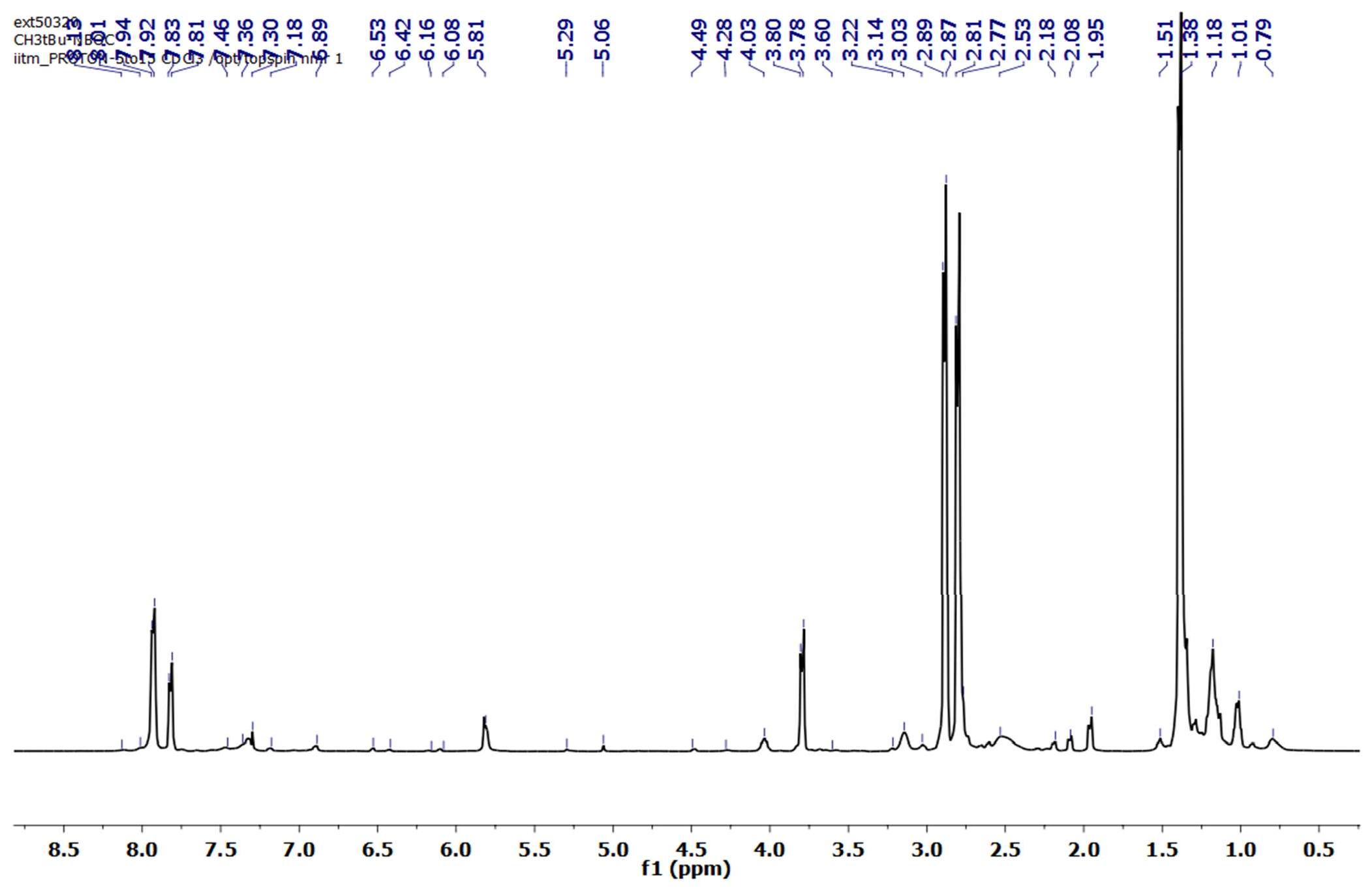


**Step 4: BOC-deprotection**


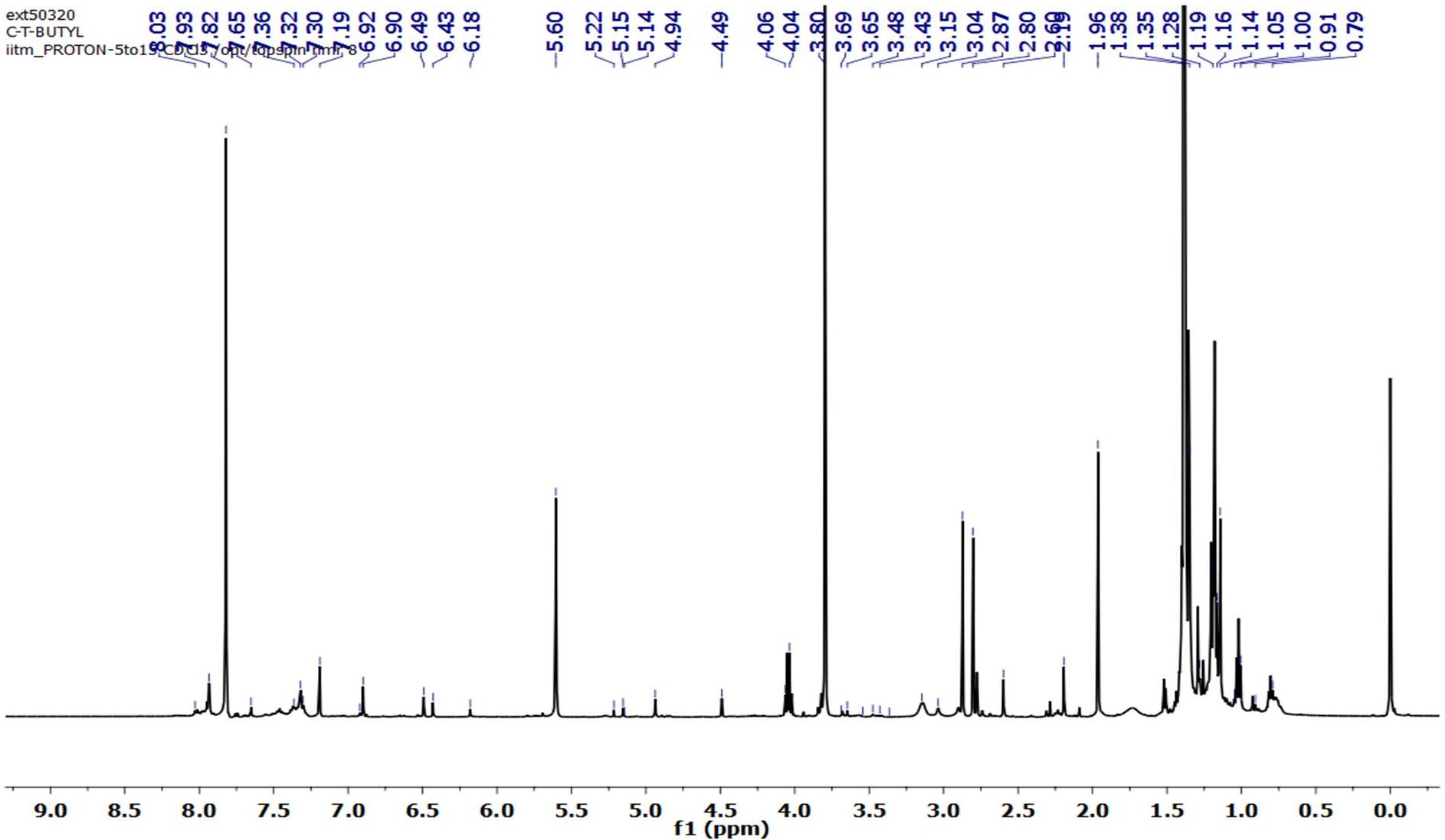

Supplement: S1 File — (DOCX) [file pone.0282454.s001.docx]
