# Supplementary material for: In-vivo studies on Transitmycin, a potent Mycobacterium tuberculosis inhibitor
Source: PLoS One. 2023 Mar 3;18(3):e0282454. doi: 10.1371/journal.pone.0282454 (PMC9983862; doi:10.1371/journal.pone.0282454)
Supplement: Attachment — Submitted filename: Plos One Reviewers comments addressed by the Authors.docx [file pone.0282454.s002.docx]

Dear Sir,

We thank all the reviewers for their extensive review and comments which helped us to revise the manuscript in its best form. And personally we again thank the reviewers to have given us an opportunity to resubmit the manuscript with all the comments addressed and tabulated.

**Title: In-vivo studies on Transitmycin, a potent *Mycobacterium tuberculosis* inhibitor**

**Reviewer 1:**

We thank the reviewer for their insightful comments and suggestions, which enabled us to strengthen our manuscript in every way possible. We responded to all of the comments and revised the manuscript. We hope the current version appeals to you and would be accepted for publication.

| **S.No** | **Reviewers comments** | **Corrections made by authors** | **Current Line numbers** |
| --- | --- | --- | --- |
| 1 | Line 231, please keep the citation format consistent | The citation in that line was inappropriate and hence removed. | L237 |
| 2 | L98 I am not sure if the ratio is written correctly (100%:1%) | Changed to (99%:1%) | L102 |
| 3 | Line 272, what is exact range of <0.312 and 40, 0 is also <0.312, this way of expression is very unprofessional. | The sentence has been changed to 0.312µg/ml - 40µg/ml | L287 |
| 4 | Line 273, A maximum of 19 and 15 isolates got inhibited at a 5μg ml-1and 10μg ml-1concentration of TR respectively. Why a lower concentration has a higher number of inhibited isolated? | The content is rephrased | L294 |
| 5 | The authors did not properly assign and arrange figures and supplementary figures, I see a number of supplementary figures while only a few figures in main text | Now the figures are arranged as given in the manuscript |  |
| 6 | The authors did a terrible job in preparing figures. In figure1, what are the numbers under the X axis? What is the Y axis? What are the units? two drug susceptible isolates of M. tuberculosis, which one is the first one, which is the second? I have a difficulty in understanding this figure 1, the authors definitely need to explain more detail in the figure legend. | The figure 1 is modified, reworked and anlaysed as Fig2.tif with appropriate legends, units and has been elaborated | L290 |
| 7 | From the figure 1, if my interpretation is correct, this TR chemical is not even as efficient as kanamycin, what’s the significance of the usage of this chemical to treat TB | We have re-worked the analysis with the corresponding critical concentrations of the anti-TB drugs used and TR . Here in this work we have compared the level of drug resistance in terms of the level of the MICs of the anti-TB drugs which varies from their recommended CCs and the same is compared with that in case of TR in this modified figure. | L292 |
| 8 | Figure 2 has no units and detailed figure legend | Figure 2 is changed to Fig3.tif inserted with appropriate legend and units | L304 |
| 9 | Figure 3, at least the authors should keep the style, format, such as font and size consistent. In addition to this issue, no legend. What are G1 G2 and G3, why G2 and G3 stopped after week5 or 6? | Figure 3 is corrected and the legends are now self-explanatory. | S3 Fig |
| 10 | Line 332, the authors need to write the numbers in a more scientific format, what is the number of ~6.10 log10? The authors need to go through the entire text and figures and tables to list numbers in a scientific way. | The format of text and figures are corrected | L349 |
| 11 | Line 351, what is PK? | The abbreviation for Pk is Pharmacokinetics and this has been indicated in the text. | L365 |
| 12 | Line 357, a docking analysis is not able to confirm anything, it is a simple prediction. | Theoretical confirmation was based on several in silico experiments  not only docking and included in the manuscript | L375 |
| 13 | Line 369. Hence it could be concluded that the anti-mycobacterial activity of TR is because of its DNA intercalation property. I do not see sufficient data to conclude this, docking prediction analysis does not count. | SOM based analysis was done and is included in the manuscript | L375 |
| 14 | Line 380, the analogs interaction with DNA needs to be experimentally tested but not just by prediction. | Analog interaction with DNA was experimentally proven by EtBr displacement assay | L259 to L270 |
| 15 | Line 421, how do the authors know RecA is a target of TR, again the prediction analysis does not serve as an evidence. | High-throughput cheminformatics tools and bioinformatics methodologies are excellent tools for predicting experimental outcomes. We applied similarity search by ML methods. Yes, as the reviewer suggests that it was not proved by invitro methods. | L405 |
| 16 | There are two sets of supplementary figures and tables with this manuscript. One at the end of it and another as a supplementary file. This created a lot of confusion for me. Please combine them into one and number them correctly. A lot of up figures and table were not referenced in the manuscript. | Now there are only one supplementary information file provided with all the supplementary figures and tables arranged sequentially. | Supplementary figures given separately are renamed as supporting information as per the journals requirements. |
| 17 | Missing reference for suramin | Reference 29 was included | Ref 29 |
| 18 | Line 433, the authors do not have experimental data suggesting TR hits the above 3 targets | One of them had been tested (DNA intercalating), but the rest of them are yet to be tested invitro and will be attempted in future. |  |
| 19 | Line 590, what is supplementary figure1, please do not simply crop the image from somewhere else without even erasing extra contents. And again, the authors should learn how to write a proper figure legend to explain figure contents. | The S1 Fig has been changed and inserted into the main text as Fig 1. | L122 |
| 20 | Supplementary figure 4, 5 and 10 are redundant to main figures. | The figures are checked for its redundancy and modified |  |
| 21 | Supplementary figure 6, what are a b,c? Simply stacking figures while not explaining them will not serve well to explain your study. | The explanation for a,b,c are given in the legend | S13 Fig. |
| 22 | Supplementary figure 14, what are the organs? | The organs lung, liver and spleen are labelled | S6B Fig |

**Reviewer 2**

We thank the reviewer for their insightful comments and suggestions, which enabled us to strengthen our manuscript in every way possible. We addressed all of the comments in the revised manuscript. We hope the current version appeals to you and would be accepted for publication.

| **S.No** | **Reviewers comments** | **Corrections made by authors** | **Line number** |
| --- | --- | --- | --- |
| 1 | Please provide the source of all the chemicals and biologicals | It is included now in the revised manuscript. |  |
| 2 | There are two sets of supplementary figures and tables with this manuscript. One at the end of it and another as a supplementary file. This created a lot of confusion for me. Please combine them into one and number them correctly. A lot of supp figures and table were not referenced in the manuscript. | Now the supplementary figures and tables to be revealed to the readers are included in the supporting information file and the rest of the information for the reviewers are provided in the supplementary documents. |  |
| 3 | Please provide a figure with TR and all its analogues in the manuscript | The figure of TR is inserted as Figure 1 and figures for analogues are given in the S14 Fig. | S14 Fig |
| 4 | L127. Provide reference for broth microdilution method here | Reference 26 | L132 |
| 5 | Please provide a brief description of the LRP assay | This has been included in the revised manuscript | L153 |
| 6 | Need more description about the Pharmacokinetic study on TR method | Elaborated in the text | L158 |
| 7 | L164, Possible typo, the peritoneal route? | Mentioned in S1 Table for the experiment | L171 |
| 8 | L169, typo, tittered | Corrected to tittered | L177 |
| 9 | L198, should be supp fig 2 | Corrected S1 fig | L204 |
| 10 | L205, please refer to supp fig 3. Fig needs to be reformatted | It is reformatted | S3 Fig |
| 11 | L249, how the fluorescence was collected? Plate reader/ fluorimeter etc? | The details have been incorporated. (Multimode mode plate reader, SpincoBiotech) | L266 |
| 12 | L246, which software was used? How the analysis was done. Need more information for the methods. | The description of the tools used had been included in the manuscript | L254 to L257 |
| 13 | Fig 1 and 2 needs more information in the legend. In its current form I cannot understand what any of these figure means. What is in X and Y axes? Probably need more detail method about the experiment | the figure is changed with corrected legend and units Fig2 and Fig 3 in the revised manuscript | Fig2, Fig3 |
| 14 | Paragraph 287, Please summarize the result in a table or figure | S11 Table is now attached. | S11 Table |
| 15 | Figure 3 needs more information in the legend. What are those lines? What is G1, G2 G3? | Figure 3 is moved to S3 Fig and legends given | S3 Fig |
| 16 | L306, 308. Again wrong figure was referenced here | This is rectified and modified to S4 Fig | S4 Fig |
| 17 | L308, I think there is a figure missing here for histopathological examination | included | S4 Fig |
| 18 | L313 wrong fig referenced. Some the bar graphs have no error bars | This is rectified and can be seen in S5 Fig | S5 Fig |
| 19 | L338 wrong figure cited | This in L367 and L369; the related figures are in S6A Fig. and S6B Fig. respectively | S6A Fig and S6B Fig |
| 20 | L347, 348 only headings? | The lines have been removed from the text |  |
| 21 | L355, what kind of basic analysis showed this? | The analysis part is now incorporated. | L375 |
| 22 | L363 wrong figure | The issue is now rectified and can be seen in S7 Fig | S7 Fig, S8 Fig |

**Reviewer 3**

We thank the reviewer for their insightful comments and suggestions, which enabled us to strengthen our manuscript in every way possible. We responded to all of the comments and revised the manuscript. We hope the current version appeals to you and would be accepted for publication.

| **S.No** | **Reviewers comments** | **Corrections made by authors** | **Line number** |
| --- | --- | --- | --- |
|  | We note that the grant information you provided in the ‘Funding Information’ and ‘Financial Disclosure’ sections do not match. When you resubmit, please ensure that you provide the correct grant numbers for the awards you received for your study in the ‘Funding Information’ section. "Authors thanks the Indian Council of Medical Research for providing grant-in-aid (F.No.5/8/5/8/TF/2017/ECD-I, to conduct this preclinical study on Transitmycin". We note that you have provided funding information that is not currently declared in your Funding Statement. However, funding information should not appear in the Acknowledgments section or other areas of your manuscript. We will only publish funding information present in the Funding Statement section of the online submission form.  Please remove any funding-related text from the manuscript and let us know how you would like to update your Funding Statement. Currently, your Funding Statement reads as follows:  "The funders had no role in study design, data collection and analysis, decision to publish, or preparation of the manuscript".  Please include your amended statements within your cover letter; we will change the online submission form on your behalf. | This will be followed while uploading the revised manuscript. |  |
|  | Your ethics statement should only appear in the Methods section of your manuscript. If your ethics statement is written in any section besides the Methods, please delete it from any other section. |  | L74-L76 |
|  | Please include a copy of Tables 1 and 6 which you refer to in your text on pages 7 and 19, respectively | Tables 1 and 2 are included in the supplementary tables in the revised manuscript |  |
