# Supplementary material for: In-vivo studies on Transitmycin, a potent Mycobacterium tuberculosis inhibitor
Source: PLoS One. 2023 Mar 3;18(3):e0282454. doi: 10.1371/journal.pone.0282454 (PMC9983862; doi:10.1371/journal.pone.0282454)
Supplement: Attachment — Submitted filename: Plos One Reviewers comments addressed by the Authors.docx [file pone.0282454.s003.docx]

Dear Sir,

We thank the reviewers for their brief comments. And personally we again thank the reviewers to have given us an opportunity to include necessary insight into animal study.

**Title: In-vivo studies on Transitmycin, a potent *Mycobacterium tuberculosis* inhibitor**

Changes made in the manuscript pertaining to reviewers comments:

We had responded to the comments and revised the manuscript accordingly. We hope the revised version appeals to you and would be accepted for publication.

| **S.No** | **Reviewers comments** | **Corrections made by authors** | **Current Line numbers (without track changes)** |
| --- | --- | --- | --- |
| 1 | Provide the details of the humane end points experimental procedures | Animal ethical approval was obtained for conducting the study | L248- L260 |
|  | In particular please provide more clarity on the 6 experimental groups used | Paragraphs on experimental groups are included | L231-244 |
|  | Rationale for performing the infection/efficacy study in Figure S2 and specify the animals died before meeting the criteria for euthanasia. | Paragraphs included | L374-L388 |
|  | Provide a survival curve for this study | Fig4. Plot of Kaplan-Meier survival analysis /Survival curve is included in the figures | L377 |
|  | L240 | Reference included [31] | L644-L649 |
|  | L253 | Reference included [32] | L650-L652 |

Thanks and regards,

Dr. Azger Dusthackeer
